# Mobile and Self‐Sustained Data Storage in an Extremophile Genomic DNA

**DOI:** 10.1002/advs.202206201

**Published:** 2023-02-03

**Authors:** Fajia Sun, Yiming Dong, Ming Ni, Zhi Ping, Yuhui Sun, Qi Ouyang, Long Qian

**Affiliations:** ^1^ Center for Quantitative Biology Peking University 5 Yiheyuan Road Haidian District Beijing 100871 P. R. China; ^2^ Academician Workstation of BGI Synthetic Genomics BGI‐Shenzhen Huada Comprehensive Park Yantian District Shenzhen 518083 P. R. China; ^3^ The State Key Laboratory for Artificial Microstructures and Mesoscopic Physics Peking University 5 Yiheyuan Road Haidian District Beijing 100871 P. R. China

**Keywords:** biomaterials, DNA data storage, error correction codes, genome engineering, nanopore sequencing

## Abstract

DNA has been pursued as a novel biomaterial for digital data storage. While large‐scale data storage and random access have been achieved in DNA oligonucleotide pools, repeated data accessing requires constant data replenishment, and these implementations are confined in professional facilities. Here, a mobile data storage system in the genome of the extremophile *Halomonas bluephagenesis*, which enables dual‐mode storage, dynamic data maintenance, rapid readout, and robust recovery. The system relies on two key components: A versatile genetic toolbox for the integration of 10–100 kb scale synthetic DNA into *H. bluephagenesis* genome and an efficient error correction coding scheme targeting noisy nanopore sequencing reads. The storage and repeated retrieval of 5 KB data under non‐laboratory conditions are demonstrated. The work highlights the potential of DNA data storage in domestic and field scenarios, and expands its application domain from archival data to frequently accessed data.

## Graphical abstract

In this study, a data storage system in *Halomonas bluephagenesis* genomes, which supports frequent and rapid data retrieval and automated data regeneration in non‐professional environments, are developed. This system expands the application scenarios of DNA data storage to frequent “warm data” accessing in household and field settings, and also underscores the potentials of living cells and long‐fragment DNA for data storage.

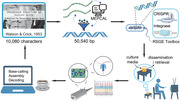

## 1 Introduction

**Figure 1:**
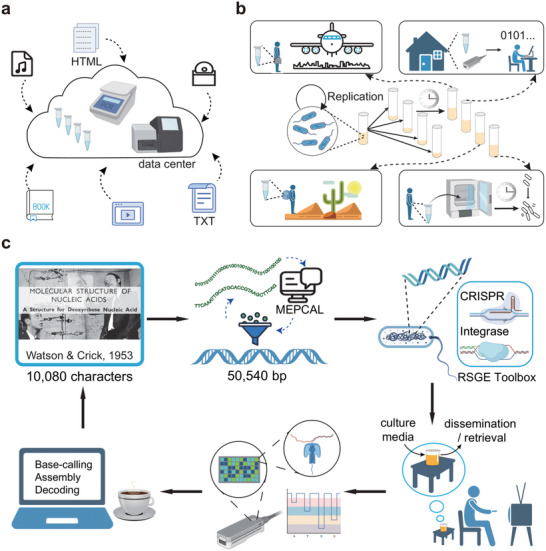
Digital information storage with bacterial genomic DNA and information retrieval using nanopore sequencing. a) Schematic of centralized DNA data storage, as realized in oligo pools. b) Schematic of genomic data storage system, which is convenient for data transfer and retrieval, and sustainable for data distribution and regeneration. c) Work flow of genomic data storage system. Digital information was first compressed and encoded by MEPCAL to generate information DNA. Next, the RSGE toolbox was used to integrate long DNA fragments into bacterial genomes. Information DNA automatically replicated as cells proliferated under indoor environment. For information retrieval, a portable MinION sequencer was used for real‐time sequencing, and a laptop was used for data processing. After base‐calling and assembly, erroneous information DNA was decoded by MEPCAL to perfectly restore the original information.

The astronomically growing global data sphere has posed an imminent challenge to data storage technologies.^[^ ^1^, ^2^ ^]^ Modern data storage systems employ a stratified structure in which data is categorized by the access frequency, and stored in different media or computational layers accordingly.^[^ ^3^, ^4^ ^]^ In the pursuit of novel storage materials, DNA has shown significant potential for the storage of “cold” data that are infrequently accessed. Gigabyte‐scale data storage in a DNA oligo pool and addressed retrieval of minute fractions of data have been demonstrated,^[^ ^5^, ^6^, ^7^, ^8^, ^9^, ^10^, ^11^, ^12^, ^13^, ^14^, ^15^ ^]^ and massively parallel synthesis and sequencing technologies are substantiating the vision of DNA‐based data centers for gigantic cold data archiving (Figure **1**a). In contrast, “warm” and “hot” data are medium‐scale data in circulation. These data are frequently distributed and accessed on‐premises, at home and en route (**Figure** **1**b). Although DNA oligo pools can be extremely portable and the pocket‐size MinION nanopore sequencer enables rapid data readout,^[^ ^16^, ^17^ ^]^ data sustainability remains problematic for the frequent access demand of warm data. In oligo pools, data is statically stored in that once sampled, the DNA is not automatically replenished. Consequently, a finite number of retrievals may lead to data exhaustion for small files in a large archive.^[^ ^11^, ^14^ ^]^ The engagement of the polymerase chain reaction (PCR) provides a solution but at the cost of accumulating systematic data errors and biases.^[^ ^18^ ^]^ In addition, the requirement for a professional PCR setup jeopardizes the mobility of the in vitro oligo storage system.

Live cells with active DNA replication work as mini Xerox machines for “data” stored in their genomes. Therefore, storage of digital data in intracellular amplicons presents a general model of self‐sustained DNA data storage. For example, large scale data storage has been demonstrated on an artificial chromosome^[^ ^19^ ^]^ (38 KB) and on plasmids in a bacterial population^[^ ^20^ ^]^ (445 KB). However, genetic instability and copy number fluctuations of these exo‐genomic amplicons ultimately impact data integrity.^[^ ^21^, ^22^ ^]^ In comparison, the genome provides a relatively stable storage environment. Currently, studies integrating DNA containing digital data into bacterial genomes relied on the CRISPR technology and thereby were limited to a few bits of information.^[^ ^23^, ^24^, ^25^ ^]^ Moreover, previous studies were mostly done in the model organisms *Escherichia coli* and *Saccharomyces cerevisiae* in sterile and growth‐controlled laboratory environments. Recently, Qian et al. used spores of *Bacillus subtilis* carrying DNA barcodes for real‐world object tracking as in IoT applications,^[^ ^26^ ^]^ suggesting that harnessing environmentally robust microbes for digital data storage may greatly expand its applicational realms.

In this work, we propose a mobile and self‐sustained storage system based on long artificial DNA in the genome of *Halomonas bluephagenesis* (Figure 1c). As a non‐model organism, this halophilic bacterium is regarded as a potential chassis for portable and open fermentation due to its unique features of anti‐contamination and easiness of cultivation and cell collection.^[^ ^27^, ^28^, ^29^, ^30^, ^31^, ^32^ ^]^ This feature is exploited here for mobility, that is, prolonged data storage and frequent data retrieval achieved in nonlaboratory environments with minimal professional handling. Digital data is genomically stored in tens of kilobases (kb) of continuous synthetic DNA for both stability and dosage control and for a maximized storage density. The MinION sequencer is employed for real‐time data readout.^[^ ^33^, ^34^ ^]^

The technical challenges of developing the system are twofold. The first challenge is large scale genomic integration. Although several strategies are available for the genomic integration of large synthetic DNA in model organisms *E. coli* and yeast, such as the *λ*‐Red system, nucleases, integrases, or their combinations,^[^ ^35^, ^36^, ^37^, ^38^ ^]^ in most other industrial bacteria, these techniques are still developing.^[^ ^39^, ^40^, ^41^ ^]^ In particular, no such system has been reported in *H. bluephagenesis*. The second challenge is readability. Despite a few attempts, restoring information from noisy nanopore reads with ∼10% errors rich in insertions and deletions (indels) has remained a daunting task.^[^ ^12^, ^42^, ^43^, ^44^ ^]^ Current indel correcting codes either rely on consensus voting, which requires substantial sequencing coverage, or are expensive in computation time. These features significantly limiting the storage capacity and the retrieval speed.^[^ ^10^, ^45^ ^]^ Another work circumvents this issue by coding with short sequences of distinctive nanopore output signals, but this approach strongly constrains the sequence space available for coding.^[^ ^16^ ^]^

To solve these problems, we designed a genetic toolbox and a coding scheme. The genetic toolbox enables genomic integration of 10–100 kb‐scale DNA fragments in *H. bluephagenesis*. The coding scheme efficiently targets indels without compromising storage density. The two were combined to establish a prototypic genomic storage system that aims to extend the territory of DNA storage from cold data to warm data, and make it available in non‐professional facilities.

## 2 Results and Discussion

### 2.1 System Construction and Stability Evaluation

We selected the seminal article revealing the double helical DNA structure by Watson and Crick for genomic storage.^[^ ^46^ ^]^ The article, a 5.56 KB text file, was encoded to two DNA sequences of lengths 29 and 51 kb (termed information DNA) by different strategies. The information DNA was synthesized by commercial company and delivered in the form of plasmids containing DNA fragments of 3–8 kb in length. These DNA fragments were first assembled into continuous fragments with a length of 12–18 kb, and then iteratively integrated into the genomes of *E. coli* and *H. bluephagenesis* by a recombinase‐based site‐specific genome engineering (RSGE) toolbox we developed (Note S1, Supporting Information). Among the integrases that have been shown to work in *E. coli*, very few had been proven functional in a different species.^[^ ^47^, ^48^, ^49^, ^50^, ^51^ ^]^ Through a comprehensive screen, we obtained 16 integrases that successfully worked in *H. bluephagenesis* (Table S1, Supporting Information), with each recognizing a specific pair of attB and attP sites with extraordinary sequence specificity.

**Figure 2:**
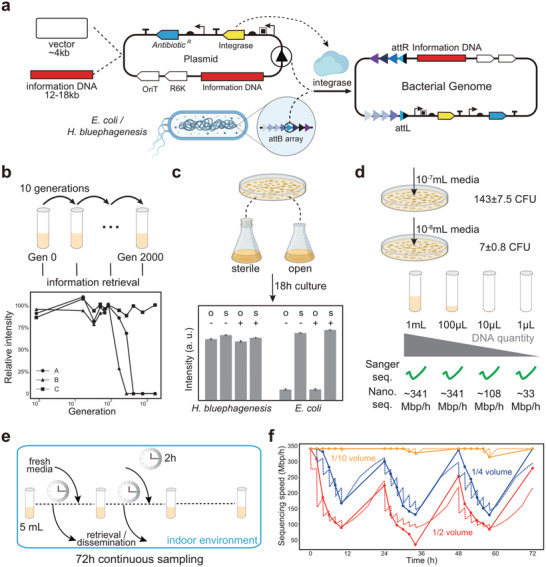
Construction and characterization of the genomic data storage system. a) Schematic of integration of information DNA into bacterial genomes using the RSGE toolbox. b) Bacterial passaging experiments. Chart below: the intensities of PCR bands of strains cultured without antibiotics relative to that of strains cultured with antibiotics. A, B, and C refer to three non‐overlapping ∼1 kb regions covering the junctions between the information DNA and the genome. The intensities of the strain cultured in antibiotic‐containing media kept steady and close to those of the genomic control (Figure S3, Supporting Information). c) Open culture of information‐bearing strains. Chart below: the intensities of PCR bands of *E. coli* and *H. bluephagenesis* after 18 h culture in different conditions (Figure S4, Supporting Information). O, open; S, sterile. Data were shown as mean ± SEM of *n* = 20 regions (∼2 kb each) collectively covering the entire information DNA. d) Colony counting and readability. Different volumes of saturated culture media were used for colony counting and information retrieval with Sanger sequencing and nanopore sequencing. Numbers for nanopore sequencing indicate sequencing speeds. e) Continuous sampling scheme of a desktop DNA information storage system. f) MinION sequencing speeds of extracted genomic DNA at each sampling point. Colored numbers indicated the sampling volume. At each point,100 µL of culture media was used for the speed test. Dots and solid lines were experimental results; dotted lines were model predictions (Note S4, Supporting Information).

To integrate information DNA into bacterial genomes, we employed a landing pad strategy. A “receiver cassette” containing 16 attB sites was inserted in *E. coli* and *H. bluephagenesis* genomes by CRISPR‐mediated homologous recombination via a dual plasmid system (Figure S1, Supporting Information). Next, we assembled the synthesized DNA fragments with plasmid vectors via Golden Gate assembly,^[^ ^52^ ^]^ resulting in plasmids carrying an integrase gene and its corresponding “sender” attP site, as well as information DNA of lengths 12–18 kb. The plasmids were then transformed into host cells. Upon transformation, the expressed integrase promoted integration of information DNA at its corresponding attB site within the receiver cassette (**Figure** **2**a). Two and three rounds of transformation were conducted for information DNA coded by two strategies to generate *H. bluephagenesis* and *E. coli* carrying up to 51 and 29 kb of continuous synthetic DNA, respectively. No evident decrease in doubling time was observed for the information‐bearing strains, albeit the growth of these cells was slightly affected depending on the length of integrated information DNA (Movie S, Figures S2a and S5, Supporting Information).

In engineered host genomes, long tracts of synthetic DNA are often prone to fragmental loss and translocation, as well as spontaneous mutations. Therefore, during a passaging experiment lasting 2000 generations, Sanger sequencing was performed at seven time points for overlapped fragments covering the entire information DNA. Among the above‐mentioned genetic errors, only one base substitution was observed in the cells at 100th generation (Table S8, Supporting Information). Antibiotics were found to be dispensable within a limited culture time (∼100 generations). Fragmental DNA loss was observed during an extended culture period without antibiotics (Figure 2b). *H. bluephagenesis* thrives in high salt environments where most bacteria undergo growth arrest. The advantage of this resistance to biological contamination in data storage was illustrated when we cultured the information‐bearing *H. bluephagenesis* strain under 6% w/v NaCl condition in either sterile or open environments for 18 h. In both experiments, the intact information DNA was successfully retrieved by PCR at the endpoint. In contrast, information DNA stored in *E. coli* was not retrievable after 18 h of open culture under the regular 1% saline condition (Figure 2c).

### 2.2 Frequent and Rapid Data Retrieval from a Benchtop Storage System

In contrast to data storage in oligo pools, cell growth offers automatic data regeneration from loss incurred by long‐term storage and frequent retrievals. We first tested how the genomic storage system withstood host dormancy, which represented a long‐term storage scenario. The information‐bearing *H. bluephagenesis* was allowed to grow until saturation in a high‐salt LB medium. The saturated culture medium was then mixed with equivoluminal 50% glycerin and placed in a −20 °C refrigerator. After frozen for 14 months, the mixture was thawed at room temperature and 1:100 diluted into fresh medium. The bacteria regained its maximal density after overnight incubation in a shaker. The saturated culture medium (OD ≈ 1.0) contained ∼10^9^ CFU mL^−1^ bacteria as extrapolated from serial dilution and colony generation experiments (Figure 2d). We tested the sequencing speed of exceedingly small samples loaded on the MinION sequencer. Unamplified genomes extracted from one milliliter of saturated culture medium resulted in a sequencing speed of ∼340 Mbp h^−1^, which would allow for information recovery in a 10‐min sequencing time (see Section 4). When the sample volume was reduced by 1000‐fold (1 µL), the sequencing speed decreased by only ∼tenfold (Figure 2d). Notably, the full process of information retrieval, including genome extraction, library construction, sequencing, assembly, and decoding, was completed within one to a few hours (Note S2, Supporting Information).

Next, we designed a continuous sampling scheme to challenge the system with recurrent information retrievals (Figure 2e). The revived *H. bluephagenesis* culture medium was placed on a bench, uncovered and unshaken (i.e., a household setting). During a 72‐h period, sampling was done every 2 h for a total of six times every day. At each sampling timepoint, certain volumes of the master culture medium (2.5 mL/1.25 mL/0.5 mL from a 5 mL pool) were taken for sequencing, and the same volumes of the fresh medium were replenished. The bacteria, along with the information DNA in their genomes, slowly replicated and regained quantity during sampling intervals. The recovery behavior was fitted to a bacteria growth model that serves to predict the availability of information given more frequent retrievals or larger sampling volumes (Note S3, Supporting Information). In our experiments, all sampling schemes supported recurrent data readout at nearly the maximal sequencing speed. To probe a scenario where cell availability would affect sequencing speeds, we reduced the culture medium sampling volume to 100 µL, and then extracted DNA for sequencing. The largest variations in sequencing speed (∼15‐fold reduction) were between the first and the sixth samples each day for the 2.5 mL group. This was consistent with the cell density estimates by the model (Figure 2f).

### 2.3 Coding Strategy

Compared to data storage in oligo pools, the cellular storage system poses unique coding challenges. First, sequences with potential biological activities (e.g., recognition sites of enzymes and recombination elements) may induce host interactions. Second, nanopore sequencing generates indels and context‐specific error profiles at high rates. An ideal coding scheme should be able to handle significant coding constraints while be efficient at correcting indel errors. To this end, we designed an error correction code named Mixed Error Processing Coding for Arbitrary Length (MEPCAL).

**Figure 3:**
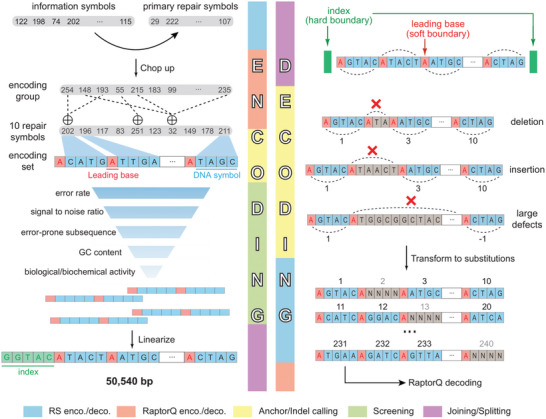
Algorithm pipeline of MEPCAL. Left: Encoding. First, the compressed information along with the primary repair symbols generated by RS code were divided into encoding groups. Next, secondary repair symbols were generated using RaptorQ code for each encoding group. These symbols were then transformed into DNA sequences (encoding set), in which a leading base was added before every DNA symbol. These DNA sequences were screened using five sequential filters. Screened sequences were concatenated and appended with tertiary RS repair symbols (not shown), and interval indices were added between sequences. Finally, sequences of encoding groups were concatenated, with interval indices added between sequences, generating the final information DNA. Right: decoding. Information DNA was first divided in accordance to the preset interval indices. Indel calling was then performed for each encoding set to restore potential DNA symbols, with each symbol endowed a serial number based on its position in the sequence. Vacant serial numbers were considered as erasures, and conflicting serial numbers were judged by maximal likelihood. DNA symbols and their corresponding serial numbers (unique mapping) were then sent to the RS decoder and finally the RaptorQ decoder.

MEPCAL employed a layered structure combining nested Reed‐Solomon (RS) code,^[^ ^53^ ^]^ RaptorQ code,^[^ ^54^ ^]^ and an anchoring approach. In this study, its processing unit was base‐256 numbers (information symbols), which was directly convertible to 4‐bp DNA symbols. The MEPCAL encoder proceeded in four steps (**Figure** **3**). First, information symbols were generated from the original information and appended by RS repair symbols at 8.6% redundancy rate before they were partitioned into encoding groups. Second, each encoding group was further organized into 16 encoding sets (packets), from which RaptorQ code, a variant of the fountain code, was applied to generate a surplus of repair packets. Regardless of their origin, the same number of packets would suffice for the restoration of the encoding group (Note S4, Supporting Information), providing sufficient coding flexibility at minimal redundancy cost. In Step 3, all packets were transcoded to DNA sequences, and a designated “leading base” was inserted between DNA symbols. These leading bases served as anchors for indel detection in the decoding process. Packets were then subjected to five filters to reject sequences with high error rates, low signal‐to‐noise ratios in the nanopore ionic current streams, extremely error‐prone sub‐sequences, inappropriate GC ratios and potential biological activities (potential open reading frames (ORFs), RSGE recombination sites, repetitive sequences, Golden gate exicision sites, etc.). These filters were customizable to the specific sequencing platform, the host organism and the genetic cloning method, to collectively enhance stability in DNA synthesis and transformation, and reduce error rates ab initio in sequencing. Next, from all packets, 16 qualified sequences were selected, and RS code was applied again at 50% redundancy rate to provide the second level of error protection. Finally, all symbols were organized linearly according to the order of encoding sets and then encoding groups and indexed by 5‐bp and 10‐bp interval indices, respectively. Through the MEPCAL encoder, the compressed 5.56 KB text data^[^ ^55^ ^]^ was coded into a DNA sequence of 50 540 bp (0.886 bit/base), of which 27.87% was for RS redundancy, 18.04% was for leading bases, and 9.77% was for indexing.

The decoding process of MEPCAL included 1) segmentation: locating the interval indices between encoding groups and encoding sets by a maximal likelihood algorithm, 2) indel calling: identifying DNA symbols in each encoding set to infer length variations due to indels, 3) RS decoding of substitutions and erasures, and 4) RaptorQ decoding. During indel calling, each encoding set was aligned to a regularly spaced pattern of leading bases through a parsimony algorithm. Expansions and contractions of leading base spacings were identified as indels and treated as erasures of the affected symbols (Figure 3). As a result, data corruption due to a miscalled indel was locally confined, and was extremely unlikely to propagate outside the encoding set.

In parallel, we encoded the compressed file with a binary Bose–Chaudhuri–Hocquenghem (BCH) code^[^ ^56^, ^57^ ^]^ at 1.552 bit/base (28 672 bp DNA). This coding scheme neither performs ab initio error reduction nor corrects indels (Section 4). As BCH code can be regarded as a binary version of RS code, it targets substitution errors at the same rate as RS code does.

### 2.4 Nanopore Sequencing and Information Retrieval

**Figure 4:**
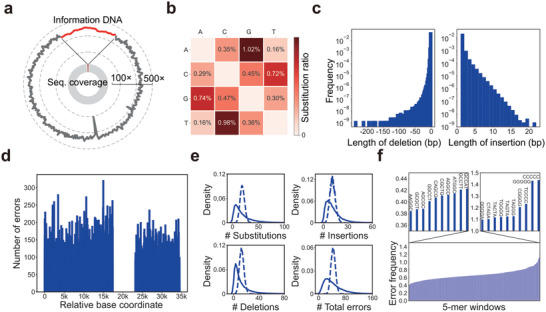
Analysis of error patterns in nanopore sequencing. a) Per‐site sequencing coverage of the bacterial genome with information DNA integrated. Data in the whole genome were smoothed with 1000‐bp windows. Data in the region of information DNA were magnified and labelled in red. b) Matrix of base substitution frequencies. The error rate (*i*, *j*) indicated the frequency of the *i*‐th base being replaced by the *j*‐th base. c) Length distribution of insertions and deletions. d) Per nucleotide error frequency along the information DNA. e) Error distribution in d for substitutions, insertions, deletions, and total errors, respectively. Dotted line in each subgraph was the Poisson distribution with the same mean. f) 5‐mer context‐dependent error frequencies. The above two subgraphs showed the ten 5‐mers with the lowest and highest error frequencies, respectively.

To obtain sufficient data for the analyses of nanopore error patterns and MEPCAL's error correction capacity, we performed ligation‐sequencing of the revived *H. bluephagenesis* strain for a prolonged period on MinION. 388 849 reads were obtained with an average read length 6.5 kb and average coverage of 603×. Per‐base coverage was uniform across the reference sequence (information DNA and the genomic backbone) (**Figure** **4**a), and all reads mapped to continuous tracts on the reference (Figure S11c, Supporting Information). Both results confirmed the genetic stability of integrated information DNA.

A close examination of raw reads from the BCH‐encoded strain revealed the error patterns of nanopore sequencing, including biased base substitution rates (Figure 4b) and indel length distributions (Figure 4c). The total error frequency was 14% at the nucleotide level, with indel rates around 7.8%. For comparison, next generation sequencing (Hi‐seq, BGI‐Shenzhen) of the same strain yielded a total error frequency of 0.58% dominated by substitutions (Table S9, Supporting Information). Non‐random and context‐dependent error profiles in nanopore reads were observed (Figure 4d), which were characteristics of the flowcell and the base‐calling algorithm employed (Note S5, Supporting Information). Specifically, right‐skewed distributions of per‐site error frequencies indicated error hotspots as verified by the 5‐mer context analysis (Figure 4e,f). These error patterns served as references for Filters 1 and 3 of the MEPCAL pipeline. Consequently, MEPCAL‐encoded information DNA exhibited significantly fewer sequencing errors than the backbone genomic sequence (*p* < 0.01, Kolmogorov–Smirnov test), with a 20–30% reduction in indel frequencies (**Table** **1**).

**Table 1:** Error statistic of nanopore sequencing results of bacteria with MEPCAL‐encoded information DNA integrated

Statistical scope	Number of reads	Number of bases	Number of substitutions	Number of insertions	Number of deletions	Number of errors
Whole genome	353 748	2 419 277 016 bp	92 730 369 bp (3.83%)	81 648 812 bp (3.37%)	97 455 858 bp (4.03%)	271 835 039 bp (11.24%)
Encoding region	6908	33 947 519 bp	1 196 299 bp (3.52%)	867 027 bp (2.55%)	1 090 817 bp (3.21%)	3 154 143 bp (9.29%)
Subsample of non‐encoding region	3831	23 653 240 bp	928 623 bp (3.93%)	836 552 bp (3.54%)	962 690 bp (4.07%)	2 727 865 bp (11.53%)

**Figure 5:**
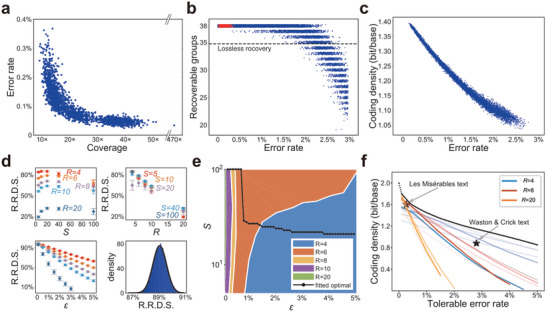
Information retrieval using MEPCAL. a) Scatter plot of the error rate of the consensus sequence versus the assembly coverage. b) The number of recoverable encoding groups under different error rates. Red dots represented the assembled sequences (data in a); blue dots represented simulated sequences with random errors added. Dots were slightly vertically shifted to show density. Dotted line indicated the minimum number of encoding groups required for perfect information retrieval. c) Effective coding density under different error rates, with bases not used for decoding excluded from the calculation. d) Dependency of the ratio of recoverable DNA symbols (R. R. D. S.) on *S*, *R*, and *ε* in MEPCAL. Identical colors indicated the same values of *S* and *R*. Data points were plotted as mean ± standard error of 2000 simulations (20000 DNA symbols for each simulation). The subgraph at the bottom right showed the distribution of R. R. D. S. when R=4, S=10, and *ε* = 0.02. The black line was the best‐fit Gaussian distribution. e) Parameter selection of *S* and *R* that maximizes the coding density under different error rates. f) Maximal coding density under different error rates (black line). Colored lines represented the density‐robustness trade‐off for combinations of different *S* and *R*. For each *R*, the value of *S* was 5, 10, 20, 40, and 100. Deeper colors indicated larger *S*.

We used Flye^[^ ^58^ ^]^ for the de novo assembly of the complete information DNA sequence. Consensus sequences with different assembly coverages were constructed from randomly sampled reads of the encoding region. The minimum assembly coverage capable of generating non‐gapped consensus was 9.13× with a post‐assembly error rate of ∼0.3%. The error rate steeply decreased and rested at ∼0.035% from 30× coverage and on (**Figure** **5**a). Each of the constructed consensuses was successfully decoded by the MEPCAL decoder to restore the original information (red dots in Figure 5b). In comparison, the consensus sequence of BCH‐encoded strain exhibited ∼0.5% total errors at 375 × coverage. Although the BCH redundancy was sufficient to correct substitutions, decoding failed due to the existence of ∼0.35% indel errors.

Given a sequencing speed of 340 Mbp h^−1^ and a host genome size of 4.2 Mbp, reads sufficient for lossless information retrieval (∼10 × coverage) can be obtained in <10 min. To probe MEPCAL's error correcting capability beyond the assembly limit, we added pseudorandom errors (with equivalent fractions of substitutions, insertions, and deletions) to the information DNA sequence to create pseudo‐sequences with 0% to 3% errors. Remarkably, information was perfectly restored from any sequence with an error rate <1.8%, while up to 2.8% errors were tolerable (Figure 5b), suggesting the potential of combining MEPCAL with less accurate but faster assembly algorithms.^[^ ^59^ ^]^

### 2.5 A Trade‐Off between Coding Density and Robustness

With the current algorithmic parameters, simulated data indicated a roughly linear decay of the effective coding density in the error rate range of 0.25–3% (Figure 5c). We developed a model to quantify the trade‐off between density and robustness, and to determine the optimal combination of parameters in MEPCAL under different error rates (Note S6, Supporting Information). Briefly, the coding density of MEPCAL is

(1) $$d=\frac{2}{1+I_{g}}\times d_{N}\times d_{E}\times \frac{S}{S+I_{s}}\times \frac{R}{R+1}$$

bit/base, where *I*_g_ and *I*_s_ are the lengths of interval indices between encoding groups and encoding sets, respectively; *d*_N_ and *d*_E_ are fractions of the primary and the tertiary repair symbols, respectively; *S* is the number of DNA symbols in each encoding set, and *R* is the length ratio of DNA symbols to leading bases.

For decoding, RS code possesses definite error correction capability, namely, information is recoverable if $Pr\left(\bar{r}\geq d_{E}\right)\geq d_{N}$, where $\bar{r}$ is the proportional difference between correctly and incorrectly recovered DNA symbols (Note S6, Supporting Information). $\bar{r}$ critically depends on *S*, *R*, and the post‐assembly error rate *ε*, namely, how well the leading base alignment algorithm locates indels and how well the mis‐alignments are constrained within the encoding set. Simulating this part of decoding, we found that $\bar{r}$ followed approximately a normal distribution N(f(S,R,ε),g(S,R,ε)), where the mean *f* and the variance *g* ^2^ were fitted from simulated data (Figure 5d). This generated an envelope curve demarcating the upper bounds of coding density and the corresponding parameter selection regimes (Figure 5e,f).

To examine the validity of the model and the performance of MEPCAL on larger data sizes, we encoded the classic novel “Les Misérables” (4.2 MB text) by Victor Hugo with the optimized parameter combination (*S*, *R*, *d*_N_, *d*_E_) deduced from the model to cope with an error rate of 0.25–0.35%, yielding a DNA sequence of 21.7 Mbp with a coding density of 1.6 bit/base (Note S7, Supporting Information). Decoding of erroneous pseudo‐sequences resolved up to 0.28% of mixed errors (Figure S15, Supporting Information), suggesting a coverage of 10–20× in nanopore sequencing was sufficient for error‐free information retrieval. The deviation from the envelope curve in Figure 5f came from a small chance of misidentification of interval sequences and leading bases.

## 3 Discussion and Conclusion

While implementing data storage in DNA pools through massively parallel DNA synthesis and next generation sequencing is essential to meet the challenge of large scale data storage, data writing and reading are carried out on physically large platforms with professional protocols to achieve high throughputs. This condition makes the frequent access of relatively small amounts of data from a civilian environment unhandy and uneconomical. Therefore, it is indispensable to develop lightweight and mobile storage systems with handy data operations (replication, distribution, retrieval, etc.) to supplement the mainstream DNA pool strategy. In this work, we reported a genomic DNA storage system supporting frequent information retrieval and distribution while being portable and self‐sustainable. The system enabled 1) durable data storage in two host growth modes (dormant and thriving), 2) automatic data regeneration on a benchtop with resistance to contaminations, 3) rapid data retrieval within one to a few hours, and 4) error‐free decoding of nanopore reads at <10 × sequencing coverages. These features point to applications of a read‐only storage model of medium storage lifespan, medium data volume, and frequent data accessing.

Two storage modes were demonstrated: an active mode for data regeneration and a dormant mode for long‐term storage. In the active mode, our 100‐day passaging experiment suggested the genomic environment of bacteria provided information stability and error‐proof information replication, considering that bacterial genomic mutation rates are 10^−9^–10^−10^/(bp·generation),^[^ ^60^ ^]^ while commercial PCR enzymes produce errors at 10^−5^–10^−6^ /(bp·cycle).^[^ ^61^ ^]^ In our experiment, the dormant mode, that is, when the bacteria were refrigerated, was tested for 14 months, and the data‐carrying strain successfully rebooted to assume the active mode in one day. Both modes and their transition were applicable in household settings. In particular, maintaining the active mode required only a normal container and culture medium supplements. The high‐salt growth condition essentially ensures the thrive of the data‐carrying host in spite of environmental microbial contaminants. The robust sequencing performance in Figure 2d,f likely spares the data retrieval protocols from sub‐milliliter liquid handlings. Storage time was also enhanced by antibiotic selection, as is commonly used in current cellular storage studies.^[^ ^19^, ^20^, ^22^ ^]^ In antibiotic‐free media, our genomic storage system preserved the entire information for 100 generations (4–5 days). In comparison, previous studies using the yeast artificial chromosome for data storage showed fragmental loss of DNA (in antibiotic media) and loss of entire YACs (in antibiotic‐free media).^[^ ^20^, ^22^ ^]^ This suggested that with or without antibiotics, bacterial genome provides a more stable environment for data storage than exo‐genomic elements.

It is meaningful to compare the storage capacity of the genomic storage system with an archival system of DNA oligo pools. For considerations of dilute solution, the indexing demand and a lower limit of 10 copies/100 µL for retrievability,^[^ ^14^ ^]^ 1 mL oligo solution can hold a maximum of ∼3 PB data. For 1 mL bacterial culture of the genomic storage system with the population storage model,^[^ ^20^ ^]^ assuming a 50 kb information DNA length, a minimum of 10 copies for readability and a 10^9^ CFU mL^−1^ bacterial concentration, the maximal storage capacity is ∼1 TB (Note S8, Supporting Information). The magnitude difference matches that between a commercial hard disk drive and a USB flash drive. It is possible to increase the storage capacity of the genomic storage system, as the RSGE toolbox supports sequential DNA integrations up to hundreds of kb. However, the amount of artificial DNA tolerable by a host microbe and the biological impacts of these genomic integrations await elucidation.^[^ ^62^, ^63^ ^]^ It is also of interest to investigate biases in the long‐term dynamics of a non‐clonal population. Population drift typically happens on the scale of 10^9^ generations for actively proliferating cells. Comparing this with the baseline bias derived from DNA synthesis and PCR in oligo pool storage^[^ ^18^ ^]^ will elucidate its impact on data integrity in a mixed‐population storage scenario.

Speed and robustness are vital for data retrieval. Compared to storage with 100–200 nt oligos, genomic storage with kb‐scale encoding lengths enables real‐time nanopore sequencing and enhances computational efficiency for de novo assembly (Note S9, Supporting Information). Congruously, several studies attempted to extend the length of oligos in a DNA pool.^[^ ^12^, ^17^ ^]^ Yet, in vitro maintenance and amplification of long DNA molecules face difficulties.^[^ ^64^ ^]^ To deal with enormous errors in nanopore sequencing, we developed a flexible and robust coding scheme named MEPCAL. Notably, RaptorQ was employed not for error correction but for effective error reduction ab initio given an arbitrary set of user‐defined constraints. For indel correction, Press et al. applied convolutional code and a greedy search algorithm to correct up to 3.59% errors in next generation sequencing readouts.^[^ ^45^ ^]^ However, this approach exhibited high computational complexity, and long codewords only exacerbate the problem. Chen et al. used superposition and a modified forward‐backward algorithm to cope with indels in nanopore reads at an error rate of 0.43%.^[^ ^20^ ^]^ However, the efficiency of the algorithm might have been contingent on short sporadic indel errors. Our results suggested that hierarchical grouping and base anchoring was sufficient to correct significant indel errors at low sequencing coverages without harming the coding density and decoding speed. Consistently, MEPCAL achieved a better coding density‐robustness trade‐off among existing coding strategies, and exhibited scant performance loss when scaling up to larger data sizes (**Table** **2**). While MEPCAL was initially developed to handle massive errors in long DNA fragments and nanopore sequencing, it can be easily adapted for DNA pools as well (Note S11, Supporting Information).

**Table 2:** Comparison to prior work

	Amount of information stored/KB	Coding density/(bit/base)	Storage carrier	Sequencing method	Minimum coverage for information retrieval	Resistance to indel
Church et al.Ref. [5]	674	0.633	DNA pool	Illumina	∼ 3000×	No
Goldman et al.Ref. [6]	635	0.290	DNA pool	Illumina	51×	No
Grass et al.Ref. [7]	83	0.862	Silica sphere	Illumina	372×	No
Blawat et al.Ref. [8]	22 528	0.892	DNA pool	Illumina	160×	No
Bornholt et al.Ref. [9]	151	0.226	DNA pool	Illumina	40×	No
Erlich et al.Ref. [11]	2097	1.569	DNA pool	Illumina	10.5×	No
Organick et al.Ref. [12]	205 005	0.822	DNA pool	Illumina	5×	No
	32/1.3			Nanopore	36×/80×	No
Press et al.Ref. [44]	128	0.595	DNA pool	Illumina	∼3×	Yes
Shipman et al.Ref. [24]	3.8	0.725	CRISPR array in *E. coli* genome	Illumina	>150–1580×	No
Chen et al.Ref. [19]	37.8	1.245	Artificial chromosome in *S. cerevisiae*	Nanopore	16.8×	Yes
This work	5.5	1.552	Long DNA fragment in bacterial genome	Sanger/HiSeq	MfA^a)^	No
		0.886		Nanopore	<9.13×	Yes
	4239(simulated)	1.600	/			Yes

^a)^ MfA: minimal coverage for assembly; see Note S10, Supporting Information, for details of “Coding density.”

The leading bases used in this work were a string of adenines (A). They can be replaced by pseudorandom watermarks or any patterned sequence to balance the overall GC content or to encode extra information into these redundancy bits. In the latter case, a bespoke decoding algorithm may achieve optimized efficiency. From the biological aspect, the effective operation of the RSGE toolbox in *H. bluephagenesis* suggested its versatility given the taxonomic distance between *Halomonas* and *Escherichia*. It is conceivable that diverse microbial physiology can be leveraged for specific storage needs, such as dormant states,^[^ ^26^ ^]^ rapid replication^[^ ^65^ ^]^ or non‐sterile storage (this work). Further studies are required to assess the utility of the RSGE toolbox in different species, as well as the practicality of various bacteria for information storage.

## 4 Experimental Section

### Bacterial Strains and Culturing Conditions

Strains used in this study included *H. bluephagenesis* TD01 (Genus: *Extremophile Halomonas spp*., obtained from ref. ^[^ ^66^ ^]^) and *E. coli* S17‐1 (Bluepha) and TOP10 (TRANS). With appropriate antibiotics added, sugar‐free LB medium was used for *E. coli* strains (1% peptone, 0.5% yeast extract, 1% sodium chloride, pH = 7.0), while salt‐rich LB medium was used for *H. bluephagenesis* TD01 (1% peptone, 0.5% yeast extract, 6% sodium chloride, pH = 7.0). All the above percentages are mass/volume ratio.

### Synthesis of Information DNA

Information DNA coded by BCH code was 28 672 bp in length, which was divided into 10 fragments. Information DNA coded by MEPCAL was 50 540 bp in length, which was divided into 6 fragments. All these fragments were synthesized by GENEray (http://www.generay.com.cn/). To obtain these long DNA fragments, short ologos of 80–100 nt were first synthesized and then assembled by PCR, resulting in longer DNA fragments with hundreds of nt in length. Next, these DNA fragments were further assembled by Gibson assembly and then loaded on a carrier plasmid, which was transformed into *E. coli* for proliferation. The correctness of the DNA sequence was verified by Sanger sequencing of the plasmid carrying synthesized DNA. The synthesized products were delivered in the form of plasmids and puncture bacteria (*E. coli*).

### Genomic Integration of attB Array

The sequence of 16 attB sites were artificially synthesized and then connected back and forth, forming an attB array with a length of 897 bp. Two plasmids were used to integrate attB array into bacterial genome, one of which carried *cas9* gene (pCas for *E. coli* TOP10, and pSEVA321 for *H. bluephagenesis* TD01), while another carried attB array and the gene of guide RNA (pTarget for *E. coli* TOP10, and pSEVA241 for *H. bluephagenesis* TD01). For *E. coli* TOP10, the first plasmid was transformed into competent cells, and the second plasmid was then introduced into screened strain via electro‐transformation. For *H. bluephagenesis* TD01, these two plasmids were first assembled via Gibson assembly and transformed into *E. coli* S17‐1 cells separately. Next, the first plasmid was introduced into *H. bluephagenesis* TD01 cells through conjugation, and the second plasmid was then introduced into screened strain via another conjugation.

### Genomic Integration of Information DNA

Information DNA were integrated into the genome of *E. coli* TOP10 following 2 steps. First, information DNA were assembled with the plasmid vector to form an intermediate plasmid containing the resistance gene, the integrase gene, the attP site, the replication origin site along with information DNA. Next, the plasmid was introduced into *E. coli* TOP10 via chemical transformation. Information DNA were then integrated into the genome of *E. coli* TOP10. This integration strategy was iteratively performed for twice to insert BCH‐coded information DNA (28.7 kb) into the genome of *E. coli* TOP10. The integration into the genome of *H. bluephagenesis* TD01 following 3 steps. First, information DNA were assembled with the plasmid vector to form an intermediate plasmid containing the resistance gene, the integrase gene, the attP site, the replication origin site along with information DNA. The plasmid was then transformed into *E. coli* S17‐1 competent cells, which still existed as plasmid and replicated as cell proliferated. Finally, *E. coli* S17‐1 containing the plasmid were conjugated with *H. bluephagenesis* TD01, and the plasmid was transferred from *E. coli* S17‐1 to *H. bluephagenesis* TD01 and integrated into the genome of the latter. This integration strategy was iteratively performed twice/3 times to insert BCH‐coded/MEPCAL‐coded information DNA (28.7 kb/50.5 kb) into the genome of *H. bluephagenesis* TD01.

### Determination of Growth Curve

The growth curve of bacteria with information DNA integrated was measured in shaker. Strains to be tested were cultured in liquid media and grown for 20 h under 200 rpm, 37 °C to reach the maximum cell density, which served as seed culture. For growth curve of bacteria cultured in shaker, 5 µL of seed culture was added to 5 mL of sterile fresh liquid medium and then cultured in shaker (200 rpm, 37 °C). For sterile setting, the absorbance was determined at 0, 2, 4, 5, 6, 7, 8, 9, 10,11, 12, 16, and 20 h after the dilution (Figure S2a, Supporting Information). For desktop culture system, the absorbance was determined at 0, 2, 4, 6, 8, 10, 14, 16, 18, 20, 24, 26, 28, 30, 32, and 34 h after the dilution (Figure S2b, Supporting Information). For each strain under each condition, 3 biological repeats were measured.

### Microscopic Imaging of Bacterial Growth

Bacteria with information DNA integrated were cultured in in liquid medium for 12 h under 200 rpm, 37 °C, and then 1:100 diluted and further cultured in in liquid medium for 4 h under 200 rpm, 37 °C. This bacterial media was then centrifuged under 5000 g for 1 min. Next, enriched bacterial cells were added into a microfluidic chip contains 4 channels, which was then centrifuged under 3500 g for 10 min. The centrifuged chip was then connected to 4 syringes, each of which was put into a pump. A thermostat was applied to monitor the temperature (37 °C). An inverted microscope (60 × oil immersion objective) was used for imaging, which lasted 12 h with a 15 min interval for each time of shooting.

### Passaging of Bacteria

Bacteria with information DNA integrated were cultured in liquid medium overnight under 200 rpm, 37 °C. This bacterial media was regarded as the 0th generation. The passaging was carried out every 12 h, in which 5 µL of bacterial media was added to 5 mL of sterile fresh liquid medium and grown under 200 rpm, 37 °C. Each time of passaging was considered to be 10 generations (2^10^ ≈ 1001).

### Open Culture

Bacteria with information DNA integrated were cultured in both sterile and non‐sterile conditions to confirm their resistance to biological contamination. Deep hole plate (24 holes) was used to culture the strain, with half of the holes sealed with a sterile film (Figure S4a, Supporting Information). Strains were cultured in liquid medium and grown for 20 h under 200 rpm, 37 °C. This bacterial media was then 1:1000 diluted, and then added to sealed (sterile) and open wells. TOP10 and TD01 with information DNA integrated were cultured in media with and without antibiotics, respectively. The plate was then placed in shaker (room temperature, 500 rpm) for 16 h.

### Co‐culture of Information‐Bearing Strains

Two strains with different length of information DNA integrated (strain A: 11 312 bp; strain B: 28 712 bp) were separately cultured in liquid media until saturated. Next, 200 µL of saturated culture media of strain A, 200 µL of saturated culture media of strain B and 6 mL of fresh media were mixed and placed in shaker (200 rpm, 37 °C) for 8 h. This bacterial media was regarded as the 5th generation (6400/200 = 32 = 2^5^). The subsequent passaging was carried out every 12 h, in which 5 µL of saturated media was added to 5 mL of sterile fresh liquid medium and grown under 200 rpm, 37 °C. Each time of passaging was considered to be 10 generations (2^10^ ≈ 1001). 3 biological replications were conducted along with control (strain A only and strain B only). The relative proportions of the two strains were quantified by fluorescent quantitative PCR at 5^th^, 10^th^, 20^th^, 40^th^, and 100^th^ generations.

### Continuous Sampling Experiments

Bacteria with information DNA integrated were cultured in liquid medium overnight under 200 rpm, 37 °C, and then diluted to reach OD600 = 1.0. Next, 5 mL of this medium was added into a 15 mL tube, and kept uncovered and unshaken under room temperature. Sampling was carried out at 12:00, 14:00, 16:00, 18:00, 20:00, and 22:00 every day, until 12:00 on the fourth day. At each sampling point, the absorbance of the medium was measure, and then 2.5 mL/1.25 mL/0.5 mL of medium was removed, with equal volume of fresh medium added. No extra fresh media was added to compensate the evaporation.

### Bacterial Cryopreservation and Revival

Bacteria with information DNA integrated were cultured in liquid medium overnight under 200 rpm, 37 °C, to reach the maximum cell density. 500 µL of saturated media was mixed with 500 µL 50% glycerin in a 1.5 mL Eppendorf tube. The mixture was then placed into a household refrigerator (−20 °C). The cryopreserved bacteria were kept in the refrigerator for more than 1 year. For revival, the frozen mixture was melted at room temperature, and 50 µL of melted mixture was added to 5 mL fresh media. Next, the media was cultured overnight under 200 rpm, 37 °C for recovery.

### Source Coding

Huffman code was used in the source coding step to compress the text that was encoded. The source entropy of the article encoded was *H* = 4.391511 bit/position, and the average code length of Huffman code used was *L* = 4.417229 bit/position, which was close to the upper limit of compression specified by Shannon's first theorem.

### Bose–Chaudhuri–Hocquenghem Encoding

The original information (binary numbers) was first divided into 224 groups. In each group, BCH (255,207) was used to generate 255‐bit BCH codeword from 207‐bit information symbols based on generator polynomial. Finally, 1 parity bit was added to the end of each group of BCH codeword, so that the Hamming weight (number of 1s) of codeword in each group was an even number. In this way, each group of codeword corresponded to exactly 128 bases. 224 groups of the above BCH code were used to store the information, resulting in 128 × 224 = 28 672 bp of DNA sequence.

### Mixed Error Processing Coding for Arbitrary Length Encoding

The original information (5564 bytes) was first padded into 5600 base‐256 numbers (symbols). RS code based on GF(2^12^) was used to generate 480 primary repair symbols. These symbols were re‐transformed into base‐256 numbers (6080 symbols) and then grouped into 38 encoding groups. Next, each encoding group (160 symbols) was used as information symbols of RaptorQ encoder to produce 399 times of secondary repair symbols (63840 symbols), and every 10 symbols were then grouped to form an encoding set. In each encoding set, each of the 10 encoding symbol was transformed into 4‐bp DNA and preceded by an additional “A,” which was called “leading base,” as a separation identifier, resulting in a 50‐bp DNA. These sequences were then screened in 5 consecutive steps: Error rate screening, signal‐to‐noise ratio screening, error‐prone sequence screening, GC‐ratio screening, and bio‐related screening. In each encoding group, 16 sequences passing through screening were retained, which were re‐transformed into base‐256 numbers and served as information symbols for RS code based on GF(2^8^) to generate 80 tertiary repair symbols. Every 10 symbols were then transformed into a 50‐bp DNA sequence in the same manner as described above and preceded by a 5 bp interval index. As a result, each group contained 24 55‐bp sequences with a total length of 1320 bp, which was comprised of 240 DNA symbols. Finally, a 10‐bp index was added before each group, generating information DNA of (1320 + 10) × 38 = 50 540 bp in length.

### Sequence Screening

Sequence screening was comprised of 5 steps. The first 3 screening were based on the analysis of error patterns in nanopore sequencing. Filter 1: Error rate screening. The single molecule sequencing machine used can accommodate 5 bases (i.e., every 5 adjacent bases generate an electrical signal when passing through the nanopore; the windows are overlapped, thus ideally each base corresponds to an electrical signal), thus 4^5^ = 1024 kinds of 5‐mer windows are available. Approximately 35 000 000 bp of nanopore sequencing results were applied to align with the original sequence (reference sequence) to obtain the error frequency of each 5‐mer. Each 50‐bp sequence contained 46 5‐mer windows, whose average error rate can be calculated based on the error frequency analysis. In order to determine the screening threshold, the average error rate of 10 000 random sequences with a length of 50 bp was calculated, and the threshold that caused 60% of the sequences to be discarded was defined. Filter 2: Signal‐to‐noise ratio screening. Certain different sequences produce similar electrical signals, causing base‐calling errors. In order to reduce these errors, the electrical signals generated by adjacent *k*‐mer windows were required to make evident difference. Here, a *k*‐mer model^[^ ^67^ ^]^ was used to calculate the “signal‐to‐noise ratio” of each 50‐bp sequence. In this model, the electrical signal generated by each window follows a Gaussian distribution with a determined mean and variance. Signal‐to‐noise ratio was defined as the average negative logarithm of the overlapping area of the Gaussian distributions of electrical signals generated by two adjacent windows, that is,

(2) $$R=\frac{1}{n-5}\sum \left(-\ln A\right)$$

where *R* was the signal‐to‐noise ratio and *A* was the above‐mentioned overlapping area

(3) $$A=\int_{-\infty }^{\infty }\min\left[f\left(x;\mu_{1},\sigma_{1}\right),f\left(x;\mu_{2},\sigma_{2}\right)\right]$$

*μ*_1_,*σ*_1_, and *μ*_2_,*σ*_2_ referred to the mean and variance of the Gaussian distribution of two adjacent electrical signals, respectively. In order to determine the screening threshold, the average signal‐to‐noise ratio of 10 000 random sequences with a length of 50 bp were calculated, and the threshold caused 60% of the sequences to be discarded was defined. Filter 3: Error‐prone sequence screening. Each 5‐mer window exhibited a specific error frequency. Among the 1024 windows, 64 with the highest error frequency were excluded. In other words, the 50‐bp sequence containing any of these 64 windows was discarded. Filter 4: GC ratio screening. 10 “A” were added to each 50‐bp sequence, causing the GC ratio of the sequence to deviate by 50%. Therefore, the GC ratio was screened to obtain sequences with a GC ratio of 40–60%, while other sequences were discarded. Filter 5: Bio‐related screening, all DNA sequences with potential biological activities were excluded in this step. Specifically, the biologically related constraints were set as follows: 1) Sequences with possible ORFs or their reverse complement sequences are discarded, for these sequences might initiate gene expression in cells. For this constraint we employed the ORF finder (NCBI).^[^ ^68^ ^]^ 2) Sequences with recombination sites of RSGE recombinases and their reverse complement sequences were discarded, for these sequences can easily trigger site‐specific recombination, thereby damage the integrity of information DNA. Specifically, all attB sites and attP sites in the RSGE toolbox are supposed to be absent in the information DNA. In addition, the recombination sites of four excisionases were excluded to enable the extended functionality of RSGE toolbox (e.g., excision of vector sequences after integration of information DNA, as illustrated in Figure S7, Supporting Information). All the sequences of these sites are listed in Table S1, Supporting Information. 3) Sequences with low complexity regions were discarded, for these regions usually exhibit evident repetitiveness, which were prone to recombination and fragmental DNA loss. In addition, repetitive sequences were hard to synthesize and sequence. Here, RepeatMasker^[^ ^69^ ^]^ was used as a reference to identify low complexity sequences. 4) Sequences with recognition sites of Golden Gate‐related excision enzyme were discarded. This operation was to ensure successful sequence assembly by the Golden Gate method.^[^ ^52^ ^]^ Specifically, GGTCTC for BsaI, GAAGAC for BbsI, and CGTCTC for BsmBI, as well as the reverse complements of the above sequences were excluded in the sequence of information DNA. Besides, all interval sequences inserted in the information DNA conformed to these rules.

### Mixed Error Processing Coding for Arbitrary Length Decoding

Information DNA was first divided into 38 encoding groups based on “maximum likelihood grouping” determined by interval indices. Using the same strategy, each encoding group was further divided to obtain 24 encoding sets, which served as the basic unit of recognition of DNA symbols. The first step of recognition was finding the position of all leading bases (“A”) in the sequence. Only DNA symbols with normal length (4 bp) were recognized and then given a serial number of 1–10. When DNA symbols with the same number appeared in an encoding set, the corresponding leading bases of these DNA symbols were compared, and the correct one was determined by the principle of “maximum likelihood.” The serial numbers of recognized DNA symbols were then transformed into absolute serial numbers based on the order of their encoding set in encoding group (from 1 to 240). The ordered DNA symbols were transformed into base‐256 numbers and then used for RS decoding. RaptorQ decoding was finally done, which was dispensable when the first 35 groups of information were successfully decoded by RS code.

### Nanopore Sequencing

The genome of bacteria was extracted and then processed for library construction before sequencing. The library was prepared using Ligation Sequencing Kit (Oxford Nanopore Technologies, catalog no. SQK‐LSK109) or Rapid Barcoding Kit (Oxford Nanopore Technologies, catalog no. SQK‐RBK004), which was then sequenced on MinION single‐molecule sequencing device (Oxford Nanopore Technologies, ONT) by loading certain amount of DNA sample on a flowcell (R9.4.1/R10.3). The sequencing device was operated using the bundled software, MinKNOW to monitor running status and perform base‐calling of the raw data.

### Quantitative Model and Optimal Parameter Selection

MEPCAL contains 5 major optional parameters: The number of extra encoding group(s) *G*, the number of DNA symbols in an encoding group *E*, the number of DNA symbols in an encoding set *S*, the length ratio of DNA symbols to leading bases *R*, the number of tertiary repair symbol in an encoding group *M*.

(4) $$\mathrm{MEPCAL}\;=\;\mathrm{MEPCAL}\left(G,\;E,\;S,\;R,\;M\right)$$

Other numerical attributes of MEPCAL can be inferred by the above 5 parameters. The coding density of MEPCAL was

(5) $$d=\frac{2}{1+I_{g}}\times d_{N}\times d_{E}\times \;d_{S}\times d_{R}\;\mathrm{bit}/\mathrm{base}$$

where

(6) $$d_{N}=\frac{N}{N+G}$$

(7) $$\;d_{E}=\frac{E}{E+M}$$

(8) $$\;d_{R}=\frac{R}{R+1}$$

(9) $$\;d_{S}=\frac{S}{S+I_{s}}$$

The sufficient condition for decoding was

(10) $$\sum_{i\;=\;1}^{N+G}D\left(i\right)\geq N$$

where *D*(*i*) referred to the decodable condition of RS code for each encoding group

(11) $$D\left.\left(i\right.\right)=\left\{\begin{array}{c} 1,\;if\;decodable\\ 0,\;if\;undecodable \end{array}\right.$$

The judgment of *D* depended on the acquisition of valid DNA symbols. The sufficient condition for *D*(*i*) = 1 was

(12) $$2\theta +\left(1-r-\theta \right)\leq \frac{M}{E+M}$$

or equivalently

(13) $$r-\theta \geq \frac{E}{E+M}$$

where *r* was the ratio of valid DNA symbols in each encoding group after decoding. Note that a DNA symbol was valid only when both its sequence and order (serial number) are correct. *θ* was the ratio of invalid DNA symbols in each encoding group after decoding. Invalid DNA symbols referred to DNA symbols with incorrect sequence or order. 1‐*r*‐*θ* was the ratio of unrecoverable DNA symbols in each encoding group.

(14) $$r+\theta \in \left[0,1\right]$$

In silico simulations under different combinations of parameters in MEPCAL as well as different error rates were performed to determine *r* and *θ*. Assuming that the separation of encoding groups and encoding sets was accurate, *r* and *θ* were exclusively related to the underlying ability of DNA symbol recognition in MEPCAL. Both *r* and *θ* were dependent variables of *R*, *S*, and error rate *ε*. For each combination of the above 3 independent variables, *r* and *θ* approximately followed Gaussian distribution. Let

(15) $$\bar{r}=r-\theta$$

Here $\bar{r}$ was related to *R*, *S*, and *ε*, which also followed Gaussian distribution when fixing the above 3 variables (Figure 5d)

(16) $$\bar{r}∼N\left(\mu ,\sigma^{2}\right){|}_{R=R_{0},S=S_{0},\varepsilon =\varepsilon_{0}}$$

where *µ* and *σ* were the mean and variance of the Gaussian distribution. Different combination of these 3 variables were selected and then performed 1000 simulations (2400 DNA symbols for each simulation) for each combination. The mean and variance of these samples were the best estimates (unbiased and effective) of *µ* and *σ*.

Multivariate polynomial fitting was performed to obtain a quantitative relationship between *µ*, *σ*, and *R*, *S*, *ε*, where the highest power of polynomials was 3.

(17) $$\mu =\sum_{i=0,\;j=0,k=0}^{i+j+k\leq 3}aR^{i}S^{j}\varepsilon^{k}$$

and

(18) $$\sigma =\sum_{i=0,\;j=0,k=0}^{i+j+k\leq 3}bR^{i}S^{j}\varepsilon^{k}$$

Let

(19) $$p\;=\;p\left(\bar{r}\geq \frac{E}{E+M}\right)$$

where *p* referred to probability. The sufficient condition for complete information retrieval was

(20) $$\left(N+G\right)\times p\geq N$$

Therefore, the sufficient condition for complete information retrieval in MEPCAL was

(21) $$p\left(\bar{r}\geq \frac{E}{E+M}\right)\geq \frac{N}{N+G}$$

Note that

(22) $$d_{N}=\frac{N}{N+G},\;d_{E}=\frac{E}{E+M}$$

Therefore, the sufficient condition for decoding was

(23) $$p\left(\bar{r}\geq d_{E}\right)\geq d_{N}$$

In which $\bar{r}$ follows a Gaussian distribution with known mean and variance, and *d*_E_ and *d*_N_ were constants less than 1. As a result, the trade‐off between coding density and error correction capability in MEPCAL can be summarized as

(24) $$\left\{\begin{array}{c} d=\frac{2}{1+I_{g}}\times d_{N}\times d_{E}\times \frac{S}{S+I_{s}}\times \frac{R}{R+1}\;bit/base\\ p\left(N\left(f\left(d_{R},d_{S},\varepsilon \right),g{\left(d_{R},d_{S},\varepsilon \right)}^{2}\right)\geq d_{E}\right)\geq d_{N} \end{array}\right.$$

Since

(25) $$\bar{r}∼N\left(\mu ,\sigma^{2}\right)$$

We can get

(26) $$\frac{\bar{r}-\mu }{\sigma }∼N\left(0,1\right)$$

Therefore,

(27) $$\begin{array}{ccc} p\left(\bar{r}\geq d_{E}\right) & = & p\left(\frac{\bar{r}-\mu }{\sigma }\geq \frac{d_{E}-\mu }{\sigma }\right)=1-p\left(\frac{\bar{r}-\mu }{\sigma }\leq \frac{d_{E}-\mu }{\sigma }\right)\\ & = & 1-\Phi_{0}\left(\frac{d_{E}-\mu }{\sigma }\right) \end{array}$$

where Φ_0_ was the distribution function of the standard Gaussian distribution *N*(0,1). When the error rate changed, the values of *d*_E_ and *d*_N_ changed accordingly, resulting in different maximum coding densities. Here, the authors fixed

(28) $$\frac{d_{E}-\mu }{\sigma }=-2.33$$

So that

(29) $$\max\left(d_{N}\right)=-\Phi_{0}\left(2.33\right)=0.99$$

For a specific error rate, *σ* was fixed. When *d*_R_ and *d*_S_ increased, *µ* decreased, which in turn reduced *d*_E_. Since the coding density was proportional to the product of these 3 parameters, the optimal trade‐off between them determined a highest encoding density.

### Statistical Analysis

No statistical methods were used to predetermine sample size. The experiments were not randomized. Data are not pre‐processed unless explicitly declared. The summary of statistics used in this study was shown in Table S12, Supporting Information. Data analysis and visualization were mostly performed in Anaconda3 (conda 4.10.3, https://www.anaconda.com/) using python3 language. Some standard python extension packages were used for these works, which included numpy, pandas, scipy, math, matplotlib, seaborn, re, random, pickle, Levenshtein, and pylab.

## Conflict of Interest

The authors declare no conflict of interest.

## Author Contributions

F.S., Y.D., L.Q., and Q.O. developed the initial concept. F.S. and Y.D. designed and performed the biological experiments. M.N. and Z.P. carried out the next‐generation sequencing. Y.S. processed the sequencing results of NGS. F.S. designed and developed the coding methods. F.S. analyzed the sequencing results under the supervision of L.Q. F.S., L.Q., and Y.D. wrote the manuscript. F.S., L.Q., and Q.O. edited the manuscript.

## Supporting information

Supporting Information

## Data Availability

The data that support the findings of this study are openly available in Online materials at https://bdainformatics.org/dataRepository, reference number 03.
