# Supplementary material for: Mobile and Self‐Sustained Data Storage in an Extremophile Genomic DNA
Source: Adv Sci (Weinh). 2023 Feb 3;10(10):2206201. doi: 10.1002/advs.202206201 (PMC10074078; doi:10.1002/advs.202206201)
Supplement: Supplementary file 1 — Supporting Information [file ADVS-10-2206201-s001.pdf]

ADVANCED SCIENCE
Open Access

Supporting Information

for *Adv. Sci.*, DOI 10.1002/advs.202206201

Mobile and Self-Sustained Data Storage in an Extremophile Genomic DNA

Fajia Sun, Yiming Dong, Ming Ni, Zhi Ping, Yuhui Sun, Qi Ouyang and Long Qian**

Supporting Information

Fajia Sun, Yiming Dong, Ming Ni, Zhi Ping, Yuhui Sun, Qi Ouyang, Long Qian**

F. Sun, Y. Dong, Q. Ouyang, L. Qian
Center for Quantitative Biology
Peking University
5 Yiheyuan Road Haidian District, Beijing 100871, P. R. China
M. Ni, Z. Ping, Y. Sun
Academician Workstation of BGI Synthetic Genomics
BGI-Shenzhen
Huada Comprehensive Park, Yantian District, Shenzhen 518083, P. R. China
Q. Ouyang
The State Key Laboratory for Artificial Microstructures and Mesoscopic Physics
Peking University
5 Yiheyuan Road Haidian District, Beijing 100871, P. R. China
E-mail: qi@pku.edu.cn
long.qian@pku.edu.cn

Supporting Information Text

Supplementary note 1: design and implementation of RSGE toolbox.

Recombinase-based Site-specific Genome Editing (RSGE) toolbox was developed to integrate long DNA fragments (>10kb) into bacterial genome. Although the DNA elements in the toolbox were mostly derived from *E. coli*[1,2], the toolbox was species-independent, rather than exclusively usable for *E. coli*. The complete implementation of the toolbox was comprised of 3 steps: integration of DNA cassette (attB array), integration of long DNA fragments and excision of vector sequence. The last 2 steps can be iteratively done to integrate several long DNA fragments, which are linearly arranged into the bacterial genome eventually.

Integration of attB array was performed using CRISPR[3,4], for which 2 plasmids were introduced into bacterial cells (Methods). After Cas cutting the target site, attB array was integrated into the bacterial genome through homologous recombination.

Integration of long DNA fragments were then performed in strains with attB array integrated. These fragments were assembled with a plasmid vector with attP site and integrase. The assembled plasmid was then introduced into bacterial cells, and the integrase was expressed inside the cell, which recognized corresponding attP site on the plasmid and attB site in attB array, and then triggered site-specific recombination. As a result, the plasmid carrying long DNA fragments was integrated into bacterial genome where an attB site previously located. Each integration consumed 1 attB site in the attB array, which was independent of other attB sites. In other words, the number of attB sites in the attB array was essentially the times for different integration. Therefore, the total length of DNA fragments that integrated into a single cell was supposed to be the product of the number of attB sites and the length of fragment in single integration. Currently, attB array contains 16 attB sites, and we have integrated up to ~18kb (except plasmid vector) DNA fragments in a single reaction. However, this limitation of length was derived from the process of plasmid construction, rather than the integration reaction. Considering that certain lysogenic viruses can completely integrate their genome (up to ~50 kb) into bacterial genome through a single reaction, the length of DNA fragments in a single integration in the RSGE toolbox should be expandable.

Excision of vector sequence ensured multiple integrations in a single cell, for DNA elements used for screening (*e.g.*, antibiotic genes) will continuously accumulate without excision, which sometimes caused difficulties for multiple integration. The excision system was independent of integration system, which depended on 2 specific excision sites and recombinases. Excision sites were set to be flanking the long DNA fragments (two sides) when assembling the plasmid. After the plasmid integrating into bacterial genome, another plasmid carrying gene of corresponding recombinase was then introduces into bacterial cells. Next, the expressed recombinase recognized the excision sites and excised the DNA sequence between 2 excision sites. As a result, long DNA fragments were integrated into the bacterial genome with almost no excess sequences.

A complete introduction as well as performance test of RSGE toolbox was fully described in our unpublished work, where all components in the toolbox were used or tested. In this work, we used integration components of RSGE toolbox, while for excision components, we merely selected one excision system as an illustration (Fig. S7), since the feasibility of multiple excision had been verified in the above work[1].

Supplementary note 2: time required to retrieve the information stored in the bacterial genome.

The retrieval of information stored in the bacterial genome was comprised of 5 steps: extraction of bacterial genomic DNA; construction of sequencing library; single molecule sequencing and base-calling; assembly/screening; decoding by MEPCAL.

The extraction of bacterial genomic DNA usually takes 0.5-1 hours following the manufacturers' instruction, depending on the process of breaking the cell wall. For *H. bluephagenesis* with strong cell wall, an incubation with lysozyme for 0.5h can maximize the amount of DNA extracted, albeit increasing the time required to extract the genome. For *E. coli*, only a mixing with lysate is required for breaking cell wall, thus the time for extraction is less than 1 hour.

Ligation Sequencing Kit (SQK-LSK109, Oxford Nanopore Technologies https://nanoporetech.com/) was used to construct the sequencing library before nanopore sequencing, which usually takes ~1 hour following the manufacturers' instruction. Rapid Barcoding Kit (SQK-RBK004, Oxford Nanopore Technologies https://nanoporetech.com/) was used for faster preparation, which usually takes ~10-20 minutes.

Single molecule sequencing and base-calling were simultaneously performed in MinION, but the latter was much slower (Fig. S11a). GPU-facilitated base-calling was guaranteed with another equipment, to which sequenced reads were instantly delivered, resulting in a comparable speed with sequencing. For MEPCAL, an average coverage of 10-20× was required to retrieve the original information (Fig. 5b), which took ~10-20 minutes to generate sufficient data.

Two methods can be used to obtain the information DNA out of the sequencing reads: screening after assembly, or assembly after screening. The former used the sequencing results to assemble the whole genome and then obtained the information DNA according to the context, while the latter screened to obtain the reads related to encoding region and then assembled it. We used Flye[5] for assembly. When the coverage was ~10×, the time required for the assembly of whole genome (~4.2Mbp) was ~0.5h. The information DNA can be immediately obtained after assembly since the position of information DNA in the genome is known. The latter method was faster, yet can introduce extraneous reads, thereby increasing the coverage required for assembly.

The decoding of MEPCAL followed 4 sequential steps, namely, dividing encoding groups and encoding sets, identifying DNA symbols, RS decoding and RaptorQ

decoding. For the MEPCAL-coded sequence in this work (50,540bp), the running time of the first 3 steps was ~0.716s (mean of 1,000 decoding), while the last step consumed ~3min. However, RaptorQ code was exclusively used for sequence screening, which took no part in error correction. Therefore, RaptorQ encoding/decoding can be replaced with other simpler manipulation such as addition with random numbers, which eliminates the time required for RaptorQ decoding.

The sum of the time required for the above 5 steps was the time required to recover artificial information from the bacterial genome, namely, 1.5-3 hours in general.

Retrieving larger-scale data follows also the above 5 steps, but the time required does not multiply increased. Extraction of bacterial genomic DNA and construction of sequencing library essentially take the same time as calculated above, since parallel operations can be applied. nanopore sequencing, base-calling and assembly/screening also take similar time to the above calculation given single cell is used for information storage, as in this work. If cell population is used, time required for these 2 steps depends on the ratio of encoding sequencing to genome size. However, DNA screening before sequencing will greatly improve the efficiency of this step, where molecular biology technology such as DNA origami can be applied.

Time required for MEPCAL decoding relies on not only the data amount, but also the sequencing quality (error rate), since most time is used for dividing DNA sequence, in which sequence alignments are iteratively done. Recovering DNA symbols from DNA sequence is the key of decoding, which actually takes a small part of the time. The algorithmic complexity (running time of program) of DNA symbol recognition was asymptotically linear when the amount of data increased (bigger S or bigger R). Given parameters used in this study (S=10, R=4), the speed of information decoding was ~194.67KB/s, or ~684.39MB/h, which is faster than the maximum sequencing speed of MinION. Table S11 shows the speed of information decoding under several different parameter combinations. In addition, pipelined information retrieval is feasible since decoding is independently performed among different encoding groups. In this way, single molecule sequencing, base-calling and MEPCAL decoding can be simultaneously performed to realize faster information retrieval, where cell sorting and DNA sorting are indispensable.

All the decoding time in this section was tested on a PC (Windows 10, Intel® Core^{TM} i7-10700 CPU @ 2.90 GHz, 64GB RAM).

Supplementary note 3: model for desktop culture and deduction of sequencing speed.

The absorbance (OD600) of the medium was measured during desktop culture, which should be proportional to the bacteria density. Monod's model[6] for chemostat was adapted for curve fitting. Although more complicated models were developed, Monod's

model was sufficient for describing the desktop culture system, since acquiring the number of bacteria with absolute precision is unnecessary.

The initial form of Monod's model was[6]

$$\frac{dX}{dt} = X[\mu(S) - D], \frac{dS}{dt} = D(S^E - S) - \frac{\mu(S)X}{Y}, Y = \frac{X}{S^E - S} \quad (1)$$

Where *X* was the biomass (in dry weight (DW) mass per unit of volume), *S* was the concentration of substrate. S^E was the concentration of fresh media, *D* was the dilution rate, *Y* was the yield coefficient. $\mu(S)$ referred to the transformation function of nutrients to bacteria. We transformed the equation as

$$\frac{dS}{dt} = -[\mu(S) - D](S^E - S) \quad (2)$$

And further the dimensionless from

$$\frac{dA}{dt} = f(W)A, \frac{dW}{dt} = f(W)(W - 1) \quad (3)$$

Where

$$A = \frac{X}{X_{max}}, W = \frac{S}{S_E} \quad (4)$$

And *f*(*W*) was a function of *W* since *W* was proportional to *S*, and *D* was a constant under each culture condition. By keeping only the linear terms in Taylor expansion of *f*(*W*), we got

$$\frac{dA}{dt} = (aW + b)A, \frac{dW}{dt} = (aW + b)(W - 1) \quad (5)$$

Where *a* and *b* were the parameters to be fitted. We set the fitting precision to three decimal places and got the optimized values for *a* and *b*, which minimized the loss

$$loss = \sum_t (A_{measure} - A_{predict})^2 \quad (6)$$

Where *t* indicated each sampling point. *Ameasure* was the measured absorbance, and *Apredict* was the predicted absorbance with different *a* and *b*. The fitting curve (Fig. S6b) was then plotted using these parameters.

We quantified the amount of DNA extracted from different amount of saturated culture media (OD≈1) using Qubit 3.0, which was used for calibrating the quantity of DNA extracted from different sampling point during desktop culture. The sequencing speed in Fig. 3E was then deduced from the "quantity-speed" curve, where we sequenced different amount of genomic DNA (from 1.725ng to 400ng) using MinION following SBK-004 protocol (Fig. S6a). We did not fit this curve using an explicit equation, rather, the estimated sequencing speed was obtained using interpolation. For example, given the amount of DNA in a sample (*n*) between the two samples we have sequenced (*n1*<*n*<*n2*), then its sequencing speed (*s*) was supposed to be between the corresponding sequencing speeds of these two samples (*s1*<*s*<*s2*), in particular

$$s = s_1 + (s_2 - s_1)\frac{n - n_1}{n_2 - n_1} \quad (7)$$

For samples with a DNA amount of >400ng, the sequencing speed was estimated to be equal to 400ng DNA sample (341Mbp/h), since 400ng is the recommended quantity in SBK-004 protocol, and the sequencing speed is unlikely to exceed 300-400Mbp/h for MinION[7].

Supplementary note 4: rationale of RaptorQ code.

Following LT code[8], several novel fountain codes have emerged with improved performance. The earliest modified version of LT code was called Rapid Tornado code (Raptor code)[9]. Compared with LT code, Raptor code achieved higher decoding success rate with the same decoding overhead. Besides, Raptor code was the first known fountain code capable of encoding and decoding in linear time. The better performance benefits from the fact that, LT code contains a small number of highly connected packets, which are eliminated by a pre-encoding process and a compensatory error correction mechanism introduced in Raptor code. RaptorQ code is the latest version of the Raptor code family and the most advanced fountain code to date[10].

The encoding process of RaptorQ code consists of 2 steps (Fig. S9)[10]. In the first step, the original K information symbols are padded with zeros to length L ($L>K$) and multiplied by a generator matrix $G_{\{L\times L\}}$ to yield L intermediate symbols. L comes from a table with some discrete values. The purpose of padding is to reduce the table information necessary for the encoder. The generator matrix of RaptorQ code is more complicated than that used in linear block codes and LT code, with symbols of LDPC (Low Density Parity Check Code), HDPC (High Density Parity Check) and LT code arranged in a square matrix. In the second step, XOR values of samples of the L intermediate symbols are taken to both reconstruct the K original information symbols and generate an arbitrary number of "repair symbols". Afterwards, these symbols are sent through a (erasure) channel. At the receiver end, the original information can be restored by either information symbols or repair symbols (both symbols are called "encoding symbols"), as long as sufficient encoding symbols are received. In practice, the relative number of encoding symbols required for decoding are much less than that in LT code. When encoding symbols equal to the number of original information symbols (K) are received, the probability of information recovery is 99%, and when one or two more encoding symbols are received, this probability reaches 99.99% and 99.9999%, respectively, irrespective of the original amount of information K[10].

In DNA-based data storage, the number of encoding symbols needed to be synthesized is related to the overall error rate of the synthesis-storage-sequencing process. For RaptorQ code, only a small number of repair symbols which can make up for the loss of DNA molecules are required. For purpose of comparison, the decoding failure rate δ of RaptorQ code is at most

$$\mathcal{O}\left(e^{k'}\right) \tag{8}$$

While

$$\delta \sim \mathcal{O}\left(e^{\sqrt{k'}/\sqrt[4]{K} - \ln(K)}\right) \tag{9}$$

for LT code[8], where k' is the number of additional encoding symbols, and K is the number of original information symbols.

For RaptorQ code, the limitation on the number of source blocks is 2^8, and that on the number of symbols in a source block is 56,403 currently. With the limitation on the symbol size being (2^{16}-1) Byte, RaptorQ code can process a limit of 946,270,874,880 byte (~946GB) of data for transmission[10]. These restrictions come from the fixed lengths of some internal parameters of RaptorQ code. However, they may be greatly lifted by means of informatics or biomolecular techniques, so that it will not become a barrier for large-scale DNA storage.

Supplementary note 5: analysis of error patterns of raw reads in nanopore sequencing.

Certain DNA sequences possess higher error rates than other sequences in DNA sequencing. However, nanopore sequencing exhibits distinctive error patterns, which is different from that in short-read sequencing such as Illumina[11,12]. To gain an in-depth inspection of these error patterns, we analyzed sequencing results of our strain with BCH-encoded information DNA integrated. Reads generated by flowcell R9.4.1 and R10.3 (ONT) were analyzed. Despite different error rate, no essential difference was found between the conclusions drawn from these 2 batches of sequencing results. Here we show the error patterns derived from sequencing results on R10.3 flowcell (137,254 reads, 977,967,436 bp in total), while sequencing results on R9.4.1 flowcell were used for designing MEPCAL. R9.4.1 flowcell was used to sequence MEPCAL-encoded information DNA, which was consistent with the coding design.

The total frequency of three types of sequencing errors - substitutions, insertions, and deletions - was 14% at the nucleotide level, with indel rates around 7.8% (Table S9). Error rates were observed to be independent of read lengths (Fig. S11b). In substitutions, transitions happened 2.7 times more often than transversions, confirming poor performance of nanopore sequencing at distinguishing between purines and between pyrimidines (Fig. 4b). For insertions and deletions, single-base deletions and single-base insertions occurred at approximately the same frequency. The maximum length of consecutive deletions was 236bp, while that of consecutive insertions was 22bp (Fig. 4c). These error patterns were consistent between information DNA and the genomic backbone (Fig. S12). The single-base error rates in the encoding region were significantly non-random (Fig. 4d). All three types of errors exhibited right-skewed distributions indicative of hotspots of sequencing errors (Fig. 4e). Indeed, sequences with homopolymeric runs ranked top in error frequencies in a 5-mer context analysis (Fig. 4f).

The above error patterns were characteristics of the flow cells of the nanopore sequencer and the base-calling algorithm employed. These patterns served as references for Filters 1 and 3 of the MEPCAL pipeline such that the resulting information DNA was biased against error-prone sequences. Consistently, the error rate of reads in MEPCAL-encoded information DNA turned out to be significantly lower than that of reads in the

backbone genomic sequence (p<0.01, Kolmogorov–Smirnov test). Specifically, we found 20%-30% reduction in the frequencies of insertions and deletions (Table 1).

Supplementary note 6: the trade-off between coding density and robustness of MEPCAL.

MEPCAL contains 5 major optional parameters: the number of extra encoding group(s) G, the number of DNA symbols in an encoding group E, the number of DNA symbols in an encoding set S, the length ratio of DNA symbols to leading bases R, the number of tertiary repair symbol in an encoding group M.

$$\mathrm{MEPCAL} = \mathrm{MEPCAL}(G, E, S, R, M) \tag{10}$$

Other numerical attributes of MEPCAL can be inferred by the above 5 parameters. For example, the number of encoding sets in each encoding group N_s is

$$N_s = \frac{E+M}{S} \tag{11}$$

Given that each symbol (base-k number) in the information to be encoded corresponds to exactly 1 DNA symbol, we can get

$$N = ceil(\frac{L}{E}) \tag{12}$$

where L refers to the length of the information stream and N refers to the number of encoding groups. L is not necessarily divisible by E, thus rounding is used. However, in the actual storage process, L can be filled with useful information to make it divisible by E. Therefore, in the subsequent computation, the rounding is omitted. The coding length (bp) of MEPCAL is

$$L' = (N + G) \cdot L_g \tag{13}$$

where L_g is the length of each encoding group

$$L_g = N_s \cdot (I_s + \log_4 k \cdot \frac{R+1}{R} \cdot S) + I_G = \frac{(E+M)\cdot(I_s + \log_4 k \cdot \frac{R+1}{R} \cdot S)}{S} + I_G \tag{14}$$

Here I_s is the length of interval sequence (bp) between encoding sets, and I_G is the length of interval sequence (bp) between encoding groups. These interval sequences serve as identifiers when separating encoding groups and encoding sets in decoding. The length of I_s and I_G was set to be

$$I_s = \mathrm{R} + 1 \tag{15}$$

And

$$I_G = 0.01 \cdot L_g \tag{16}$$

Therefore,

$$L' = \frac{(N+G)\cdot(E+M)\cdot(R+1+\log_4 k \cdot \frac{R+1}{R} \cdot S)\cdot 1.01}{S} \tag{17}$$

The coding density d is

$$d = \frac{L \cdot \log_2 k}{L'} = \frac{S \cdot L \cdot \log_2 k}{(N+G)\cdot\left[(E+M)\cdot(R+1+\log_4 k \cdot \frac{R+1}{R} \cdot S\right)\cdot 1.01]} \; bit/base \tag{18}$$

Note that

$$N = \frac{L}{E} \tag{19}$$

Therefore,

$$d = \frac{S \cdot N \cdot E \cdot \log_2 k}{(N+G) \cdot \left[(E+M) \cdot \left(R+1+\log_4 k \cdot \frac{R+1}{R} \cdot S\right) \cdot 1.01\right]}\ bit/base \tag{20}$$

Given that each DNA symbol in MEPCAL corresponds to 1 symbol in the information stream (for example, if the information represented by base-256 numbers, each 4-bp DNA symbol corresponds to exactly 1 number),

$$R = \log_4 k \tag{21}$$

Then

$$d = \frac{2 \cdot S \cdot N \cdot E \cdot \log_4 k}{(N+G) \cdot \left[(E+M) \cdot \left(\log_4 k+1+\log_4 k \cdot \frac{R+1}{R} \cdot S\right) \cdot 1.01\right]} = \frac{2}{1.01} \cdot \frac{N}{N+G} \cdot \frac{E}{E+M} \cdot \frac{R}{R+1} \cdot \frac{S}{S+1} bit/base \tag{22}$$

Let

$$d_N = \frac{N}{N+G}, d_E = \frac{E}{E+M}, d_R = \frac{R}{R+1}, d_S = \frac{S}{S+1} \tag{23}$$

then we get

$$d = \frac{2}{1.01} \cdot d_N \cdot d_R \cdot d_E \cdot d_S\ bit/base \tag{24}$$

which is independent of the amount of encoded data $\mathrm{L} \cdot \log_2 k$. The length of I_s and I_G can be arbitrarily set. In general case,

$$d = \frac{2}{1+I_g} \cdot d_N \cdot d_E \cdot \frac{S}{S+I_S} \cdot \frac{R}{R+1}\ bit/base \tag{25}$$

The values of d_N, d_E, d_R and d_S are independent and all less than 1. These parameters actually stand for different levels of redundancy in MEPCAL, for d_N is related to the error correction capability of primary repair symbols, and d_E is related the error correction capability of tertiary repair symbols. d_R and d_S are related to the ability to recognize valid coding symbols.

For MEPCAL, the sufficient condition for decoding is

$$\sum_{i=1}^{N+G} D(i) \geq N \tag{26}$$

where $D(i)$ refers to the decodable condition of RS code for each encoding group

$$\mathrm{D}(i) = \begin{cases} 1, if\ decodable \\ 0, if\ undecodable \end{cases} \tag{27}$$

The judgment of D depends on the acquisition of valid DNA symbols. The sufficient condition for $D(i)$=1 is

$$2\theta + (1 - r - \theta) \leq \frac{M}{E+M} \tag{28}$$

or equivalently

$$r - \theta \geq \frac{E}{E+M} \tag{29}$$

where r is the ratio of valid DNA symbols in each encoding group after decoding. Note that a DNA symbol is valid only when both its sequence and order (serial number) are correct. θ is the ratio of invalid DNA symbols in each encoding group after decoding. Invalid DNA symbols refer to DNA symbols with incorrect sequence or order. 1-r-θ is the ratio of unrecoverable DNA symbols in each encoding group.

$$r + \theta \in [0,1] \quad (30)$$

To determine *r* and *θ*, we performed *in silico* simulation under different combinations of parameters in MEPCAL as well as different error rates, where substitution, insertion and deletion were in equal numbers. Here we assumed that the separation of encoding groups and encoding sets was accurate, thus *r* and *θ* are exclusively related to the underlying ability of DNA symbol recognition in MEPCAL. Both *r* and *θ* were dependent variables of *R*, *S* and error rate *ε*. For each combination of the above 3 independent variables, *r* and *θ* approximately followed Gaussian distribution. Let

$$\bar{r} = r - \theta \quad (31)$$

Here $\bar{r}$ was related to *R*, *S* and *ε*, which also followed Gaussian distribution when fixing the above 3 variables (Fig. 5d)

$$\bar{r} \sim N(\mu, \sigma^2)|_{R=R_0,\ S=S_0,\ \varepsilon=\varepsilon_0} \quad (32)$$

Where *μ* and *σ* were the mean and variance of the Gaussian distribution. We selected different combination of these 3 variables and then performed 1,000 simulations (2,400 DNA symbols for each simulation) for each combination (Table S10). The mean and variance of these samples were the best estimates (unbiased and effective) of *μ* and *σ*.

To obtain a quantitative relationship between *μ*, *σ* and *R*, *S*, *ε*, we performed multivariate polynomial fitting for the data in Table S10, where the highest power of polynomials was 3.

$$\mu = \sum_{i=0,j=0,k=0}^{i+j+k\leq 3} aR^i S^j \varepsilon^k \quad (33)$$

And

$$\sigma = \sum_{i=0,j=0,k=0}^{i+j+k\leq 3} bR^i S^j \varepsilon^k \quad (34)$$

Let

$$p = p(\bar{r} \geq \frac{E}{E+M}) \quad (35)$$

Where *p* refers to probability. The sufficient condition for complete information retrieval is

$$(N + G) \cdot p \geq N \quad (36)$$

Therefore, the sufficient condition for complete information retrieval in MEPCAL is

$$p\left(\bar{r} \geq \frac{E}{E+M}\right) \geq \frac{N}{N+G} \quad (37)$$

Note that

$$d_N = \frac{N}{N+G}, d_E = \frac{E}{E+M} \quad (38)$$

Therefore, the sufficient condition for decoding is

$$p(\bar{r} \geq d_E) \geq d_N \quad (39)$$

In which $\bar{r}$ follows a Gaussian distribution with known mean and variance, and d_E and d_N are constants less than 1. As a result, the trade-off between coding density and error correction capability in MEPCAL can be summarized as

$$\begin{cases} d = \frac{2}{1+I_g} \cdot d_N \cdot d_E \cdot \frac{S}{S+I_s} \cdot \frac{R}{R+1} \ bit/base \\ p(N(f(d_R, d_S, \varepsilon), g(d_R, d_S, \varepsilon)^2) \geq d_E) \geq d_N \end{cases} \quad (40)$$

Since

$$\bar{r} \sim N(\mu, \sigma^2) \quad (41)$$

We can get

$$\frac{\bar{r}-\mu}{\sigma} \sim N(0,1) \quad (42)$$

Therefore,

$$p(\bar{r} \geq d_E) = p\left(\frac{\bar{r}-\mu}{\sigma} \geq \frac{d_E-\mu}{\sigma}\right) = 1 - p\left(\frac{\bar{r}-\mu}{\sigma} \leq \frac{d_E-\mu}{\sigma}\right) = 1 - \Phi_0\left(\frac{d_E-\mu}{\sigma}\right) \quad (43)$$

Where Φ_0 is the distribution function of the standard Gaussian distribution $N(0,1)$. When the error rate changes, the values of d_E and d_N change accordingly, resulting in different maximum coding densities. Here we fixed

$$\frac{d_E-\mu}{\sigma} = -2.33 \quad (44)$$

So that

$$\max(d_N) = -\Phi_0(2.33) = 0.99 \quad (45)$$

For a specific error rate, σ is fixed. When d_R and d_S increase, μ decrease, which in turn reduces d_E. Since the coding density is proportional to the product of these 3 parameters, the optimal trade-off between them determines a highest encoding density (Fig. 5f).

Supplementary note 7: parameters in MEPCAL used in this study and coding density.

$$\text{MEPCAL}(3,160,10,4,80) \quad (46)$$

was used to store the text[13] in this study, where

$$d_E = \frac{E}{(E+M)} = \frac{160}{(160+80)} = 0.667 \quad (47)$$

And

$$d_N = \frac{N}{(N+G)} = \frac{35}{(35+3)} = 0.921 \quad (48)$$

And

$$d_R = \frac{4}{4+1} = 0.8 \quad (49)$$

The coding density was

$$d = \frac{10 \cdot 5564 \cdot \log_2 256}{(35+3) \cdot \left[(160+80) \cdot \left(5 + \log_4 256 \cdot \frac{4+1}{4} \cdot 10\right) + 10 \cdot 10\right]} = \frac{445120}{505400} = 0.881 \ bit/base \quad (50)$$

In this case, the 36 padded symbol can also store useful information. The coding density counting these symbols was 0.886 bit/base.

For scale-up simulation, we used the above quantitative model to determine the optimal combination of parameters. Here we expected to deal with a mixed error rate of 0.25-0.35% (including substitutions, insertions and deletions), which corresponds to a sequencing coverage of 10-20× in single-molecule sequencing. The optimal parameters deduced from the above error rate were

$$R = 10, S = 100, d_E = 0.919 \tag{51}$$

Note that d_N was already fixed to be 0.99. Here we chose a slightly smaller d_N to prevent accidental factors from affecting the decoding

$$d_N = 0.98 \tag{52}$$

The amount of information to be stored (full text of "Les Miserables", English version) was 34,719,692 bits. For the convenience of encoding, we padded some data to make the data volume *D* be 34,720,000 bits, or

$$D = \frac{34720000\ bit}{8} = 4340000\ byte = 4238\ KB = 4.14\ MB \tag{53}$$

Since

$$d_N = 0.98 \tag{54}$$

the amount of primary repair symbols to be generated was

$$\frac{34720000\ bit * 0.02}{0.98} = 708572 bit \tag{55}$$

Again, we chose a value of 720,000 bits. The total amount of information and primary repair symbols was

$$34720000 + 720000 = 35440000\ bit \tag{56}$$

Now that the amount of information contained in each DNA symbol was

$$2\ bit \cdot 10 = 20\ bit \tag{57}$$

thus the total number of DNA symbols required was

$$\frac{35440000\ bit}{20\ bit/symbol} = 1772000\ symbol \tag{58}$$

The number of information symbols was

$$\frac{34720000\ bit}{20\ bit/symbol} = 1736000\ symbol \tag{59}$$

while the number of primary repair symbols was

$$\frac{720000\ bit}{20\ bit/symbol} = 36000\ symbol \tag{60}$$

To generate primary repair symbols, RS code based on GF(2^n) was supposed to be used, where

$$\frac{35440000}{n} < 2^n \tag{61}$$

The smallest integer satisfying the above formula was 21, which was, however, not a factor of 35440000. Therefore, GF(2^{25}) was used to generate 720000 bits of RS repair symbols from 34720000 bits of information.

After generating primary repair symbols, we chose an appropriate *E* so that the numbers of the information groups and primary repair symbol groups were both integers

$$\frac{34720000\ information\ symbols}{80000\ symbols/group} = 434\ groups \tag{62}$$

And

$$\frac{720000\ information\ symbols}{80000\ symbols/group} = 9\ groups \tag{63}$$

Therefore, each encoding group contained 80,000 bits of information, or, 4000 DNA symbols. Since each encoding set contained 100 DNA symbols (*S*=100), each encoding group was comprised of 40 encoding sets.

Since

$$R = 10 \tag{64}$$

the RS code used was based on GF(2^{20}). Besides,

$$d_E = 0.919 \tag{65}$$

therefore, the number of tertiary repair symbols in each encoding group was

$$M = \frac{4000symbols*(1-0.919)}{0.919} \approx 353 \tag{66}$$

However, *M* was supposed to be divisible by *S* (100), thus *M*=400 was chosen for encoding, which led to the usage of RS(4400, 4000).

In conclusion,

$$MEPCAL(9{,}4000{,}100{,}10{,}400) \tag{67}$$

was used to encoding the information. With the length of interval sequences set to 100bp between encoding groups, the coding density was

$$d = \frac{34720000\ bit}{\frac{[(10+1)bp*100+11]\frac{bp}{set}344\ set+100\ bp}{group}\cdot 443\ group} = 1.6\ bit/base \tag{68}$$

Supplementary note 8: storage density of DNA pools and bacterial storage system.

For DNA pools, information is first amplified via PCR, in which primers address specific sequences in the DNA strand (usually at both ends). Next, the duplicated DNA strands are sent to sequencing platform such as Illumina. Therefore, the feasibility of PCR and addressing of DNA strands dictate the storage density of DNA pools. At present, the information stored in a single DNA pool does not exceed hundreds of MB[11]. No research has revealed the upper limit of information stored in single libraries in the actual process. However, the length of index cannot grow indefinitely, and there can only be finite kinds of molecules in one library before it becomes too difficult to extract a specific sequence.

Due to technical bottlenecks in the current DNA synthesis process, most studies to date have used 150-250 bp oligos as storage units. Since DNA oligos are fully mixed in a library, a unique index needs to be assigned to each oligo encoding unique information. Given a strand length of 200 bp, when the length of the index in this sequence is *k* bp, the number of indexable molecules is 4*k*, and the number of bits used to store information is 400-2*k* per molecule. Therefore, the total storage capacity of the oligo pool is

$$C = (400 - 2k) \cdot 4^k\ bits \tag{69}$$

For purpose of information retrieval (*e.g.*, by means of PCR), a DNA library has to be prepared in dilute solution. In biology labs, the concentration of the most commonly used DNA solutions is about tens to hundreds of ng/μL, which is equivalent to about 3 × 10^{-4} mol/L (100 ng/uL nucleotides). Given a copy number for identical DNA strands of 10 for reliable PCR, the molar density is around 10^{-5} mol/L, which corresponds to a *k* of

23 and a storage density of ~3 PB/cm^3. At this point, the molar density of DNA is 2.39 × 10^{-5} mol/L.

For bacterial storage system, the cell density of saturated culture media is ~10^9 CFU/mL (Fig. 3d). In principle, information can be retrieved from the colony resulting from the proliferation of a single viable cell, albeit additional time required. Here we set the retrieval limit as 10 living cells carrying identical information. Assuming that each cell contains 50kb of information DNA with a coding density of 1.6 bit/base, we can get the storage density Db,

$$D_b = \frac{10^9\ CFU}{cm^3} \cdot \frac{1}{10\ CFU} \cdot 50000\ base \cdot \frac{1.6\ bit}{base} \cdot \frac{1\ byte}{8\ bit} = \frac{10^{12}\ byte}{cm^3} = 1\ TB/cm^3 \quad (70)$$

Supplementary note 9: comparison between NGS (Illumina) and nanopore sequencing (ONT).

For both NGS and nanopore sequencing, a complete sequencing process is comprised of library construction, sequencing and data analysis (*e.g.*, assembly). Regardless of the characteristics of DNA samples, NGS usually takes 5-6 hours for library construction using commercial kits[14], while this time for nanopore sequencing is ~1-2 hour(s) (Ligation Sequencing Kit) or ~10-20 minutes (Rapid Sequencing Kit). The running time of Illumina sequencing instruments is 1-2 days[14], while the running time of ONT sequencer (MinION) can be arbitrarily set, which produces real-time reads at a speed of 200-300 Mbp/h. The automated data analysis tool of Illumina takes 1-2 days[14], while ONT provides guppy software for base-calling and alignment within several hours. Further analysis of nanopore sequencing results, such as polishing and assembly, are guaranteed by various programs such as Flye[5], which takes hours for reads processing and assembly. The throughput of Illumina sequencer and ONT sequencer is ~0.5-1000 GB and ~1-4000GB, respectively , while the cost (per GB) of the two is comparable[12]. Importantly, the MinION device of ONT is portable, thus supports corporate and domestic usage.

The most significant differences between NGS and nanopore sequencing are the read length and the error rate. nanopore sequencing generates reads with a length of hundreds of KB, which is thousands of times than the read length of NGS, albeit with an error rate of 10-15%[12], which pose strict challenges of artificial information storage and retrieval through nanopore sequencing. MEPCAL was developed to solve this problem, so as to make full use of the read length and real-time feature of nanopore sequencing.

Supplementary note 10: coding density of prior DNA coding methods.

- Church *et al.*[15] used 54898 short nucleotides with a length of 159bp to store 5.27 Mbit of information, with a coding density of 5.27 Mbit/(54898×159) base =0.633 bit/base.
- Goldman *et al.*[16] used 153335 short nucleotides with a length of 117bp to store 5.2×10^6 bit of information, with a coding density of 5.2×10^6 bit/(153335×117)

base=0.290 bit/base.

- Grass *et al.*[17] used 4991 short nucleotides with a length of 158bp to store 83 KB of information, with a coding density of 83 KB/(4991×158) base=0.862 bit/base.
- Blawat *et al.*[18] used 900000 short nucleotides with a length of 230bp to store 22 MB of information, with a coding density of 22 MB/(900000×230) base=0.892 bit/base.
- Bornholt *et al.*[19] used 45652 short nucleotides with a length of 120bp to store 151 KB of information, with a coding density of 151 KB/(45652×120) base=0.226 bit/base.
- Erlich *et al.*[20] used 72000 short nucleotides with a length of 152bp to store 2146816 bytes of information, with a coding density of 2146816 byte/(72000×152) base=1.569 bit/base.
- Organick *et al.*[11] used 13448372 short nucleotides with a length of 158bp to store 200.2 MB of information, with a coding density of 200.2 MB/(13448372×152) base=0.822 bit/base.
- Press *et al.*[21] used 5865 short nucleotides with a length of 300bp to store information. Each strand contained 46bp of primers and a 254bp payload. Each payload contained 4 bytes (32 bit, 16bp) of non-coding region, while the remaining part comprising of information and redundancy depended on "code rate". They used 6 different code rate, from 0.166 to 0.75. Therefore, the highest coding density was 2 bit/base×[(254-16)×0.75÷300]=1.19 bit/base, and the highest coding density was 2 bit/base×[(254-16)×0.166÷300]=0.263 bit/base.

Supplementary note 11: usage of MEPCAL in data storage system based on DNA pools.

MEPCAL was developed mainly for resolving the massive errors in nanopore sequencing, regardless of the synthesis platform and the storage media. Therefore, MEPCAL can also be applied to DNA oligonucleotide pools in which information is stored in short DNA strands. In this case, the grouping strategy (division of encoding groups and encoding sets) of MCPCAL requires appropriate adjustments. Specifically, the length of encoding groups or of encoding sets should fit to the length of the DNA oligonucleotides. We here provide strategies for applying MEPCAL in DNA pools comprised of short strands (100-300 nt) and longer strands (>1 KB), respectively.

DNA pools containing short DNA strands (100-300 nt) serve as the mainstream storage medium in previous studies on DNA data storage[15-20]. In order to apply MEPCAL to these pools, each strand is supposed to correspond to an encoding set. The interval sequence for the encoding sets serves as an index/barcode for the ordering of the oligonucleotides. If the encoding sets are further grouped into encoding groups for a second round of Reed-Solomon encoding, the indices should also contain the encoding group identity. The rest of the oligonucleotide carry the information (the payload). The

length ratio of DNA symbols to leading bases, *R*, and the number of DNA symbols in an encoding set, *S*, can be then chosen according to the length of payload region. For example, let *S*=10 and *R*=8, the length of encoding set is therefore 10 × (8+1) = 90 nt. Appended by the index sequence and the primer sequence, the length of the whole DNA strand is ~150 nt. In the decoding of MEPCAL, the division of encoding groups and encoding sets is thereby replaced by sorting of DNA strands according to the index sequence. The subsequent decoding process is identical to that of a continuous DNA sequence (as illustrated in our work).

Sometimes the DNA pools contain DNA strands with longer length (for instance, specialized for nanopore sequencing). MEPCAL can be easily applied to such DNA pools, where each strand is supposed to correspond to an encoding group. In this case, the interval sequences between encoding sets are retained, while the interval sequences between encoding groups are replaced with index sequences. Specifically in this project, the payload region in each strand is 1320 nt, appended by a primer sequence and an index sequence, resulting in a strand with a length of ~1400 nt. In the decoding of MEPCAL, the division of encoding groups is replaced by sorting of DNA strands according to the index sequence, while the subsequent decoding process is identical to that of a continuous DNA sequence (as illustrated in our work).

Importantly, if Illumina sequencing is used for information retrieval, the strategies of sequence screening and parameter selecting in the encoding pipeline should be adjusted according to the error pattern of Illumina sequencing. Specifically, one would skip the signal-to-noise screening and choose a larger *R* since indel rates are much lower in Illumina Sequencing. In summary, MEPCAL can flexibly accommodate different storage platforms and media, while maintaining the coding efficiency and error correction capacity.

SI References

[1] L. Yang et al., Permanent genetic memory with >1-byte capacity. *Nat. Methods* **11**, 1261-1266 (2014).
[2] J. Fernandez-Rodriguez, L. Yang, T. E. Gorochowski, D. B. Gordon, C. A. Voigt, Memory and Combinatorial Logic Based on DNA Inversions: Dynamics and Evolutionary Stability. *ACS Synth. Biol.* **4**, 1361-1372 (2015).
[3] Y. Jiang et al., Multigene editing in the Escherichia coli genome via the CRISPR-Cas9 system. *Appl. Environ. Microbiol.* **81**, 2506-2514 (2015).
[4] A. Pickar-Oliver, C. A. Gersbach, The next generation of CRISPR–Cas technologies and applications. *Nat. Rev. Mol. Cell Biol.* **20**, 490-507 (2019).
[5] M. Kolmogorov, J. Yuan, Y. Lin, P. A. Pevzner, Assembly of long, error-prone reads using repeat graphs. *Nat. Biotechnol.* **37**, 540-546 (2019).
[6] M. Jacques, La technique de culture continue: theorie et applications (Academic Press, New York, 1950).
[7] Oxford Nanopore technologies, (ONT), “Real-time, on-demand sequencing in the palm of your hand” (Publication BR_1002(EN)_V6_25Aug2021, ONT, 2021;

https://nanoporetech.com/sites/default/files/s3/literature/MinION-Mk1C-brochure.pdf).
[8] M. Luby, “LT codes” in 43rd Annual IEEE Symposium on Foundations of Computer Science, (Vancouver, BC, 16-19 November 2002) pp. 271.
[9] A. Shokrollahi. Raptor codes. *IEEE Trans. Inform. Theory.* **52**, 2551-2567 (2006).
[10] M. Luby, A. Shokrollahi, M. Watson, T. Stockhammer, L. Minder, "RaptorQ Forward Error Correction Scheme for Object Delivery" (Internet Engineering Task Force, 2011; https://tools.ietf.org/html/rfc6330/).
[11] L. Organick et al., Random access in large-scale DNA data storage. *Nat. Biotechnol.* **36**, 242-248 (2018).
[12] S. Goodwin, J. D. McPherson, W. R. McCombie, Coming of age: ten years of next-generation sequencing technologies. *Nat. Rev. Genet.* **17**, 333-351 (2016).
[13] J. D. Watson, F. H. Crick, Molecular structure of nucleic acids; a structure for deoxyribose nucleic acid. *Nature* **171**, 737-738 (1953).
[14] Illumina, Inc., "An Introduction to Next-Generation Sequencing technology" (Publication 770-2012-008, Illumina, 2015; https://www.ramaciotti.unsw.edu.au/sites/default/files/2019-04/illumina_sequencing_introduction.pdf).
[15] G. M. Church, Y. Gao, S. Kosuri, Next-generation digital information storage in DNA. *Science* **337**, 1628 (2012).
[16] N. Goldman et al., Towards practical, high-capacity, low-maintenance information storage in synthesized DNA. *Nature* **494**, 77-80 (2013).
[17] R. N. Grass, R. Heckel, M. Puddu, D. Paunescu, W. J. Stark, Robust chemical preservation of digital information on DNA in silica with error-correcting codes. *Angew. Chem. Int. Ed. Engl.* **54**, 2552-2555 (2015).
[18] M. Blawat et al., Forward error correction for DNA data storage. *Procedia. Comp. Sci.* **80**, 1011-1022 (2016).
[19] J. Bornholt et al., “Toward a DNA-based archival storage system” in 21th ACM International Conference on Architectural Support for Programming Languages and Operating Systems, (Georgia, USA, 2-6 April, 2016) pp, 98-104.
[20] Y. Erlich, D. Zielinski, DNA Fountain enables a robust and efficient storage architecture. *Science* **355**, 950-954 (2017).
[21] W. H. Press, J. A. Hawkins, S. K. Jones, Jr., J. M. Schaub, I. J. Finkelstein, HEDGES error-correcting code for DNA storage corrects indels and allows sequence constraints. *Proc. Natl. Acad. Sci. U. S. A.* **117**, 18489-18496 (2020).
[22] R. C. Bose, D. K. Ray-Chaudhuri, On a class of error correcting binary group codes. *Information and Control* **3**, 68-79 (1960).
[23] A. Hocquenghem, Codes correcteurs d'erreurs. *Chiffers* **2**, 147-156 (1959).
[24] P. Elias, Predictive coding. *IRE transactions on information theory* **1(1)**, 16-24 (1955).
[25] J. Bonnet, P. Subsoontorn, D. Endy, Rewritable digital data storage in live cells via engineered control of recombination directionality. *Proc. Natl. Acad. Sci. U. S. A.* **109**, 8884-8889 (2012).
[26] F. St-Pierre et al. One-step cloning and chromosomal integration of DNA. *ACS Synth. Biol.* **2**, 537-541 (2013).
[27] Y. Xie, Y. Yao, V. Kolisnychenko, C. Teng, K. S. Kim, HbiF regulates type 1

fimbriation independently of FimB and FimE. *Infect. Immun.* **74**, 4039-4047 (2006).
[28] T. Akiyama, M. C. Gibson, Decapentaplegic and growth control in the developing Drosophila wing. *Nature* **527**, 375-378 (2015).
[29] J. H. Ivo et al. Using the GEMM-ESC strategy to study gene function in mouse models. *Nat. Protoc.* **10**,1755-1785 (2015).
[30] S. Brian, M. D. Jeffrey, DNA recombination with a heterospecific Cre homolog identified from comparison of the pac-c1 regions of P1-related phages. *Nucleic Acids Res.* **32**, 6086-6095 (2004).
[31] S. Emiko, N. Manabu, VCre/VloxP and SCre/SloxP: new site-specific recombination systems for genome engineering. *Nucleic Acids Res.* **39**, e49 (2011).

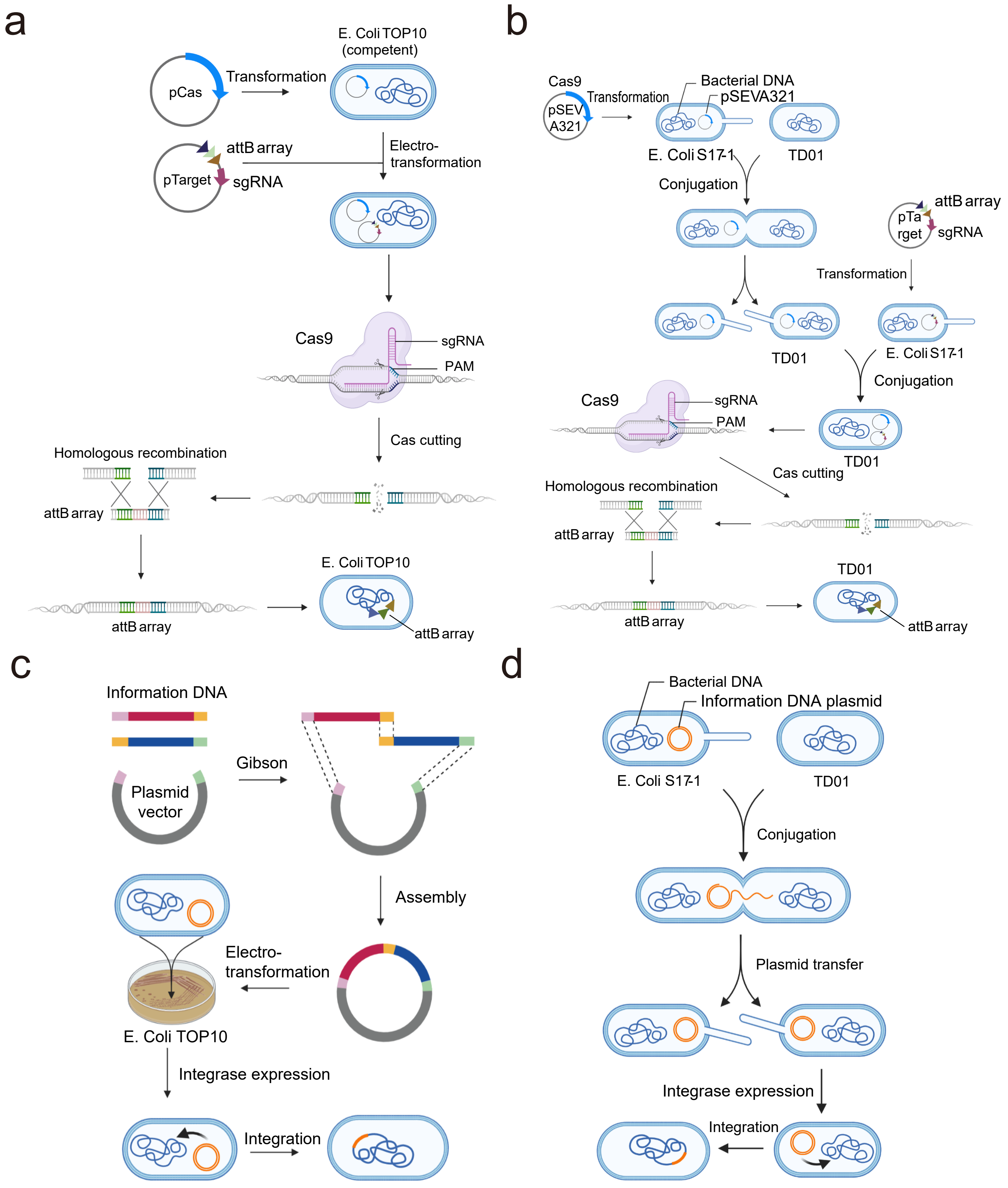

Supplementary Figure 1: Strategy of genomic integration of information DNA
a Integration of attB array into the genome of *E. coli* TOP10. **b** Integration of attB array into the genome of *H. bluephagenesis* TD01. **c** Integration of information DNA into the genome of *E. coli* TOP10. **d** Integration of information DNA into the genome of *H. bluephagenesis* TD01.

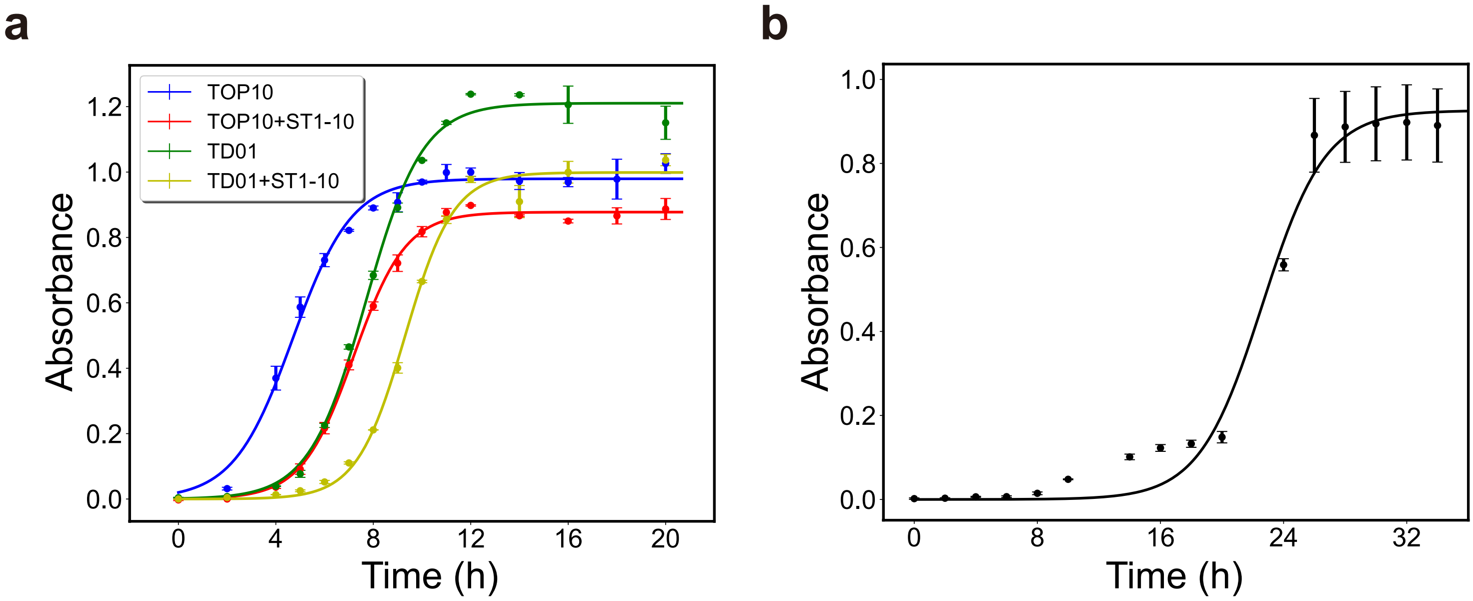

Supplementary Figure 2: Growth curve of bacteria with information DNA integrated

a Growth curve of bacte-ria cultured in shaker (37°C, 200rpm). Here "TOP10" refers to *E. coli* TOP10, and "TD01" refers to *H. bluephagenesis* TD01. "ST1-10" refers to information DNA coded by BCH code. For each strain, 3 biological replica-tions were measured at 0h, 2h, 3h, 4h, 5h, 6h, 7h, 8h, 9h, 10h, 12h, 14h, 16h, 18h (for TOP10 only) and 20h after 1:1000 dilution from overnight-cultured media. **b** Growth curve of *H. bluephagenesis* strain under indoor envi-ronment (room temperature, no cover, no shake). 3 biological replications were measured at 0h, 2h, 4h, 6h, 8h, 10h, 14h, 16h, 18h, 20h, 24h, 26h, 28h, 30h, 32h and 34h after 1:1000 dilution from overnight-cultured media. Dots indicate the mean of absorbance, and error bars indicate the standard error. Solid lines are the fitting results of 3-parameter logistic equation $A(t)=K/[1+b\cdot e^{-at}]$, where A is the absorbance; a, b, K are parameters.

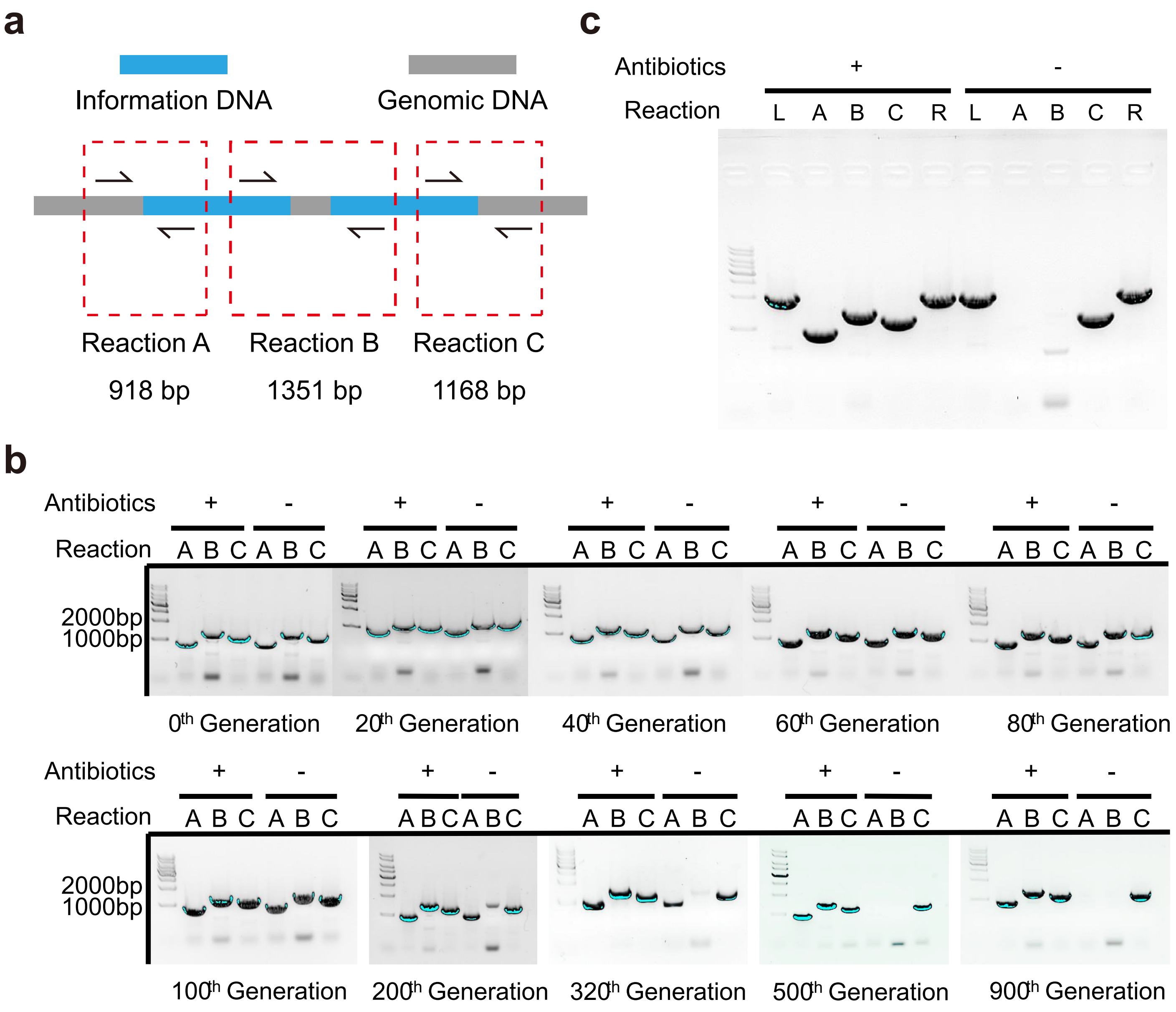

Supplementary Figure 3: PCR results of passaging of strains with information DNA integrated
a Schemat-ic of 3 PCR reactions used to verify informative DNA. These reactions spanned the junction between integrat-ed information DNA and bacterial genome. **b** PCR results of strains of 0th, 20th, 40th, 60th, 80th, 100th, 200th, 320th, 500th and 900th generation. Trans 1Kb DNA Ladder (https://www.transgen.com.cn/dna_marker/205.ht-ml) was used here to calibrate the size of PCR products. The sizes of two bands at the bottom of the DNA ladder are marked in the figure. **c** PCR results of strains of 2000th generation. L and R refer to the PCR reac-tion that amplifies the genomic sequence upstream and downstream of the information DNA, respectively.

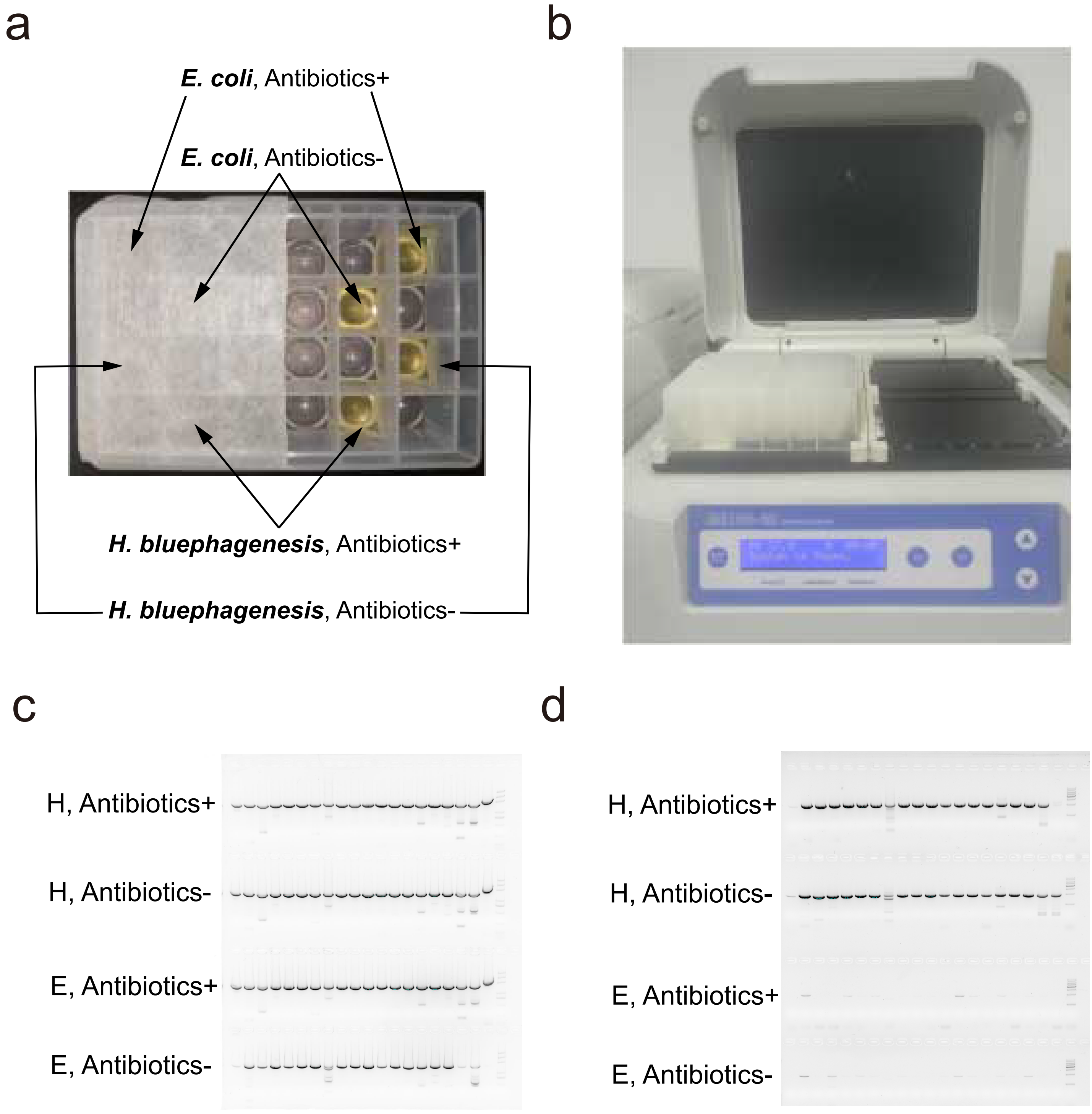

Supplementary Figure 4: Open culture of strains with information DNA integrated

Here strains of *H. bluephagenesis* TD01 and *E.coli* TOP10 with identical information DNA integrated (coded by BCH code) were used for comparison. **a** Deep-well plate for open culture. The left half of the deep-well plate was covered by a sterile film, while the right half was not covered. The integrated strains were added to the deep-well plate and then cultured under sterile (left) and open (right) environments. **b** The deep-well plate was placed in an open shaker; the culture temperature was room temperature (25 °C). The speed of shaker was set to 500rpm. **c** PCR result of strains under sterile culture condition. Each row corresponded to the strain in a hole. **d** PCR result of strains under open culture condition. Each row corresponded to the strain in a hole. H: *Halomonas bluephagenesis* TD01; E: *E.coli* TOP10.

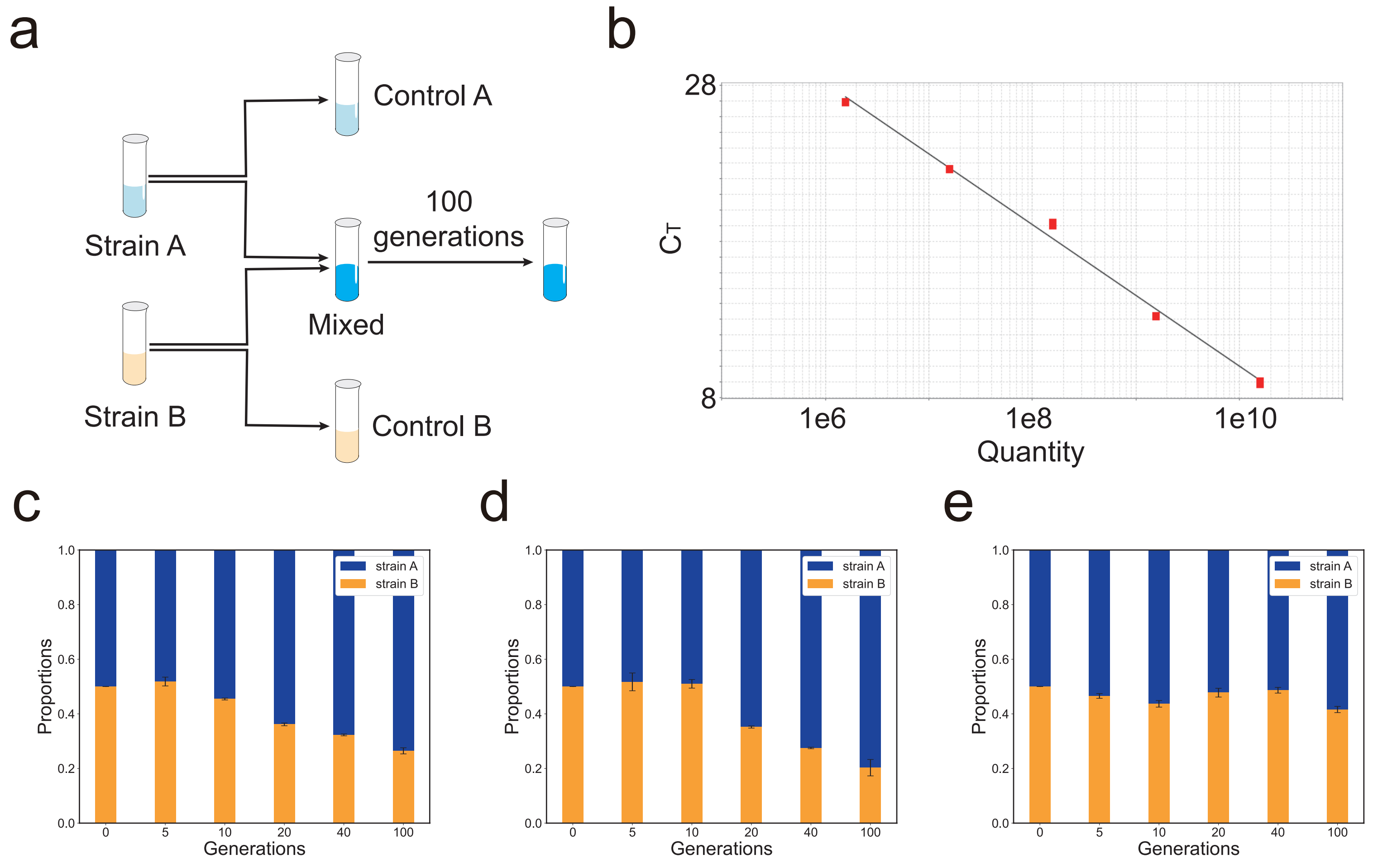

Supplementary Figure 5: Co-culture of strains with different length of information DNA integrated
a Schematic diagram of the experiment process. Strain A: 11,312 bp information DNA integrated. Strain B: 28,712 bp information DNA integrated. **b** Standard curve of the qPCR reaction. The relative proportions of the two strains of 3 biological replications quantified by qPCR are illustrated in **c**, **d**, **e**. Error bars show the mean ± standard deviation of three independent measuring.

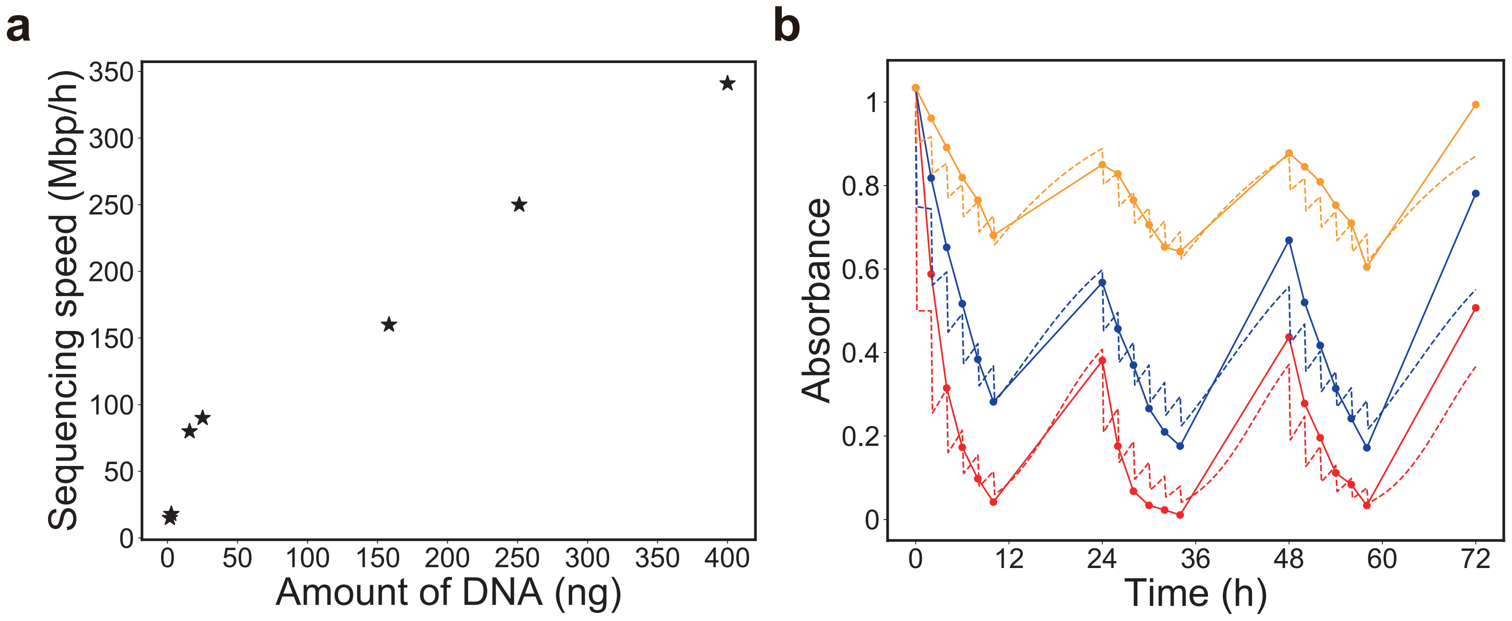

Supplementary Figure 6: Deduction of sequencing speed of desktop data storage system
a Correlation of sequencing speed of MinION with amount of DNA in sample. Each sequencing test was independent with others. All sequencing was performed following the protocol of the Rapid Sequencing Kit (SQK-RBK004) of ONT. **b** Absorbance of desktop data storage system at each sampling point. The absorbance were assumed to be propor-tional to bacterial density, and were fitted by bacterial growth model (dashed line).

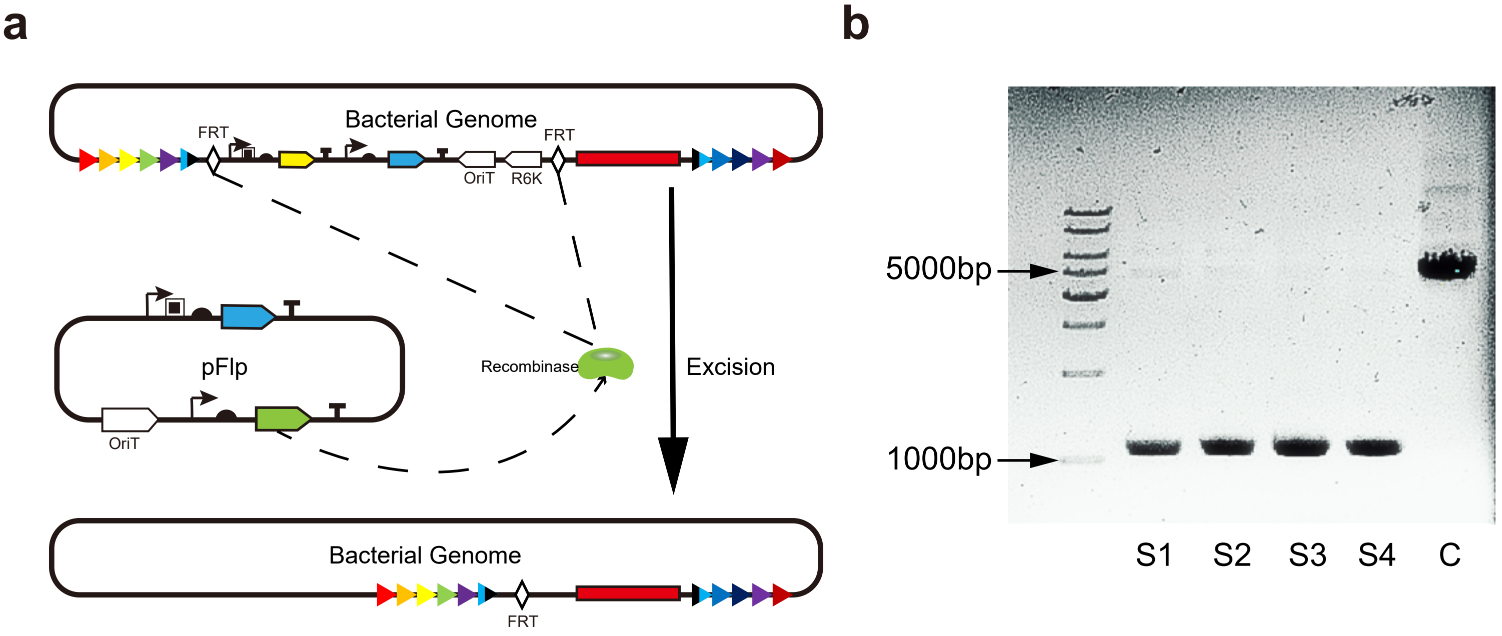

Supplementary Figure 7: Excision of plasmid vector after integration
Here strains of *E.coli* TOP10 with information DNA integrated (coded by BCH code) were used for illustration of excision. **a** Strategy of excision. **b** PCR results of excised strains (S1, S2, S3, S4 were 4 independent colonies) and untreated strain (C, refers to control).

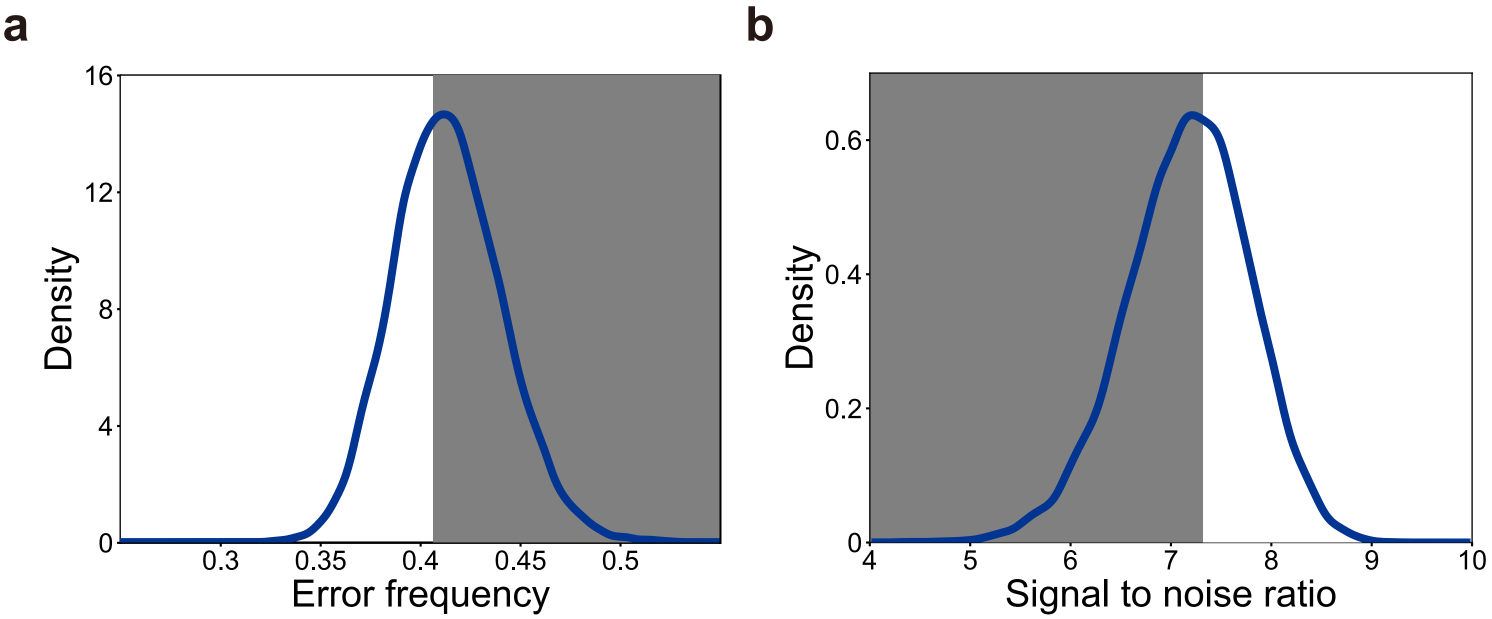

Supplementary Figure 8: Threshold for sequence screening used in this study

a Simulated data for error rate screening, where the average error rates of 46 5-mer windows in 20000 random 50-bp sequences were calculated. The threshold was determined so that 60% of the sequences were discarded (grey area). **b** Simulated data for signal-to-noise screening, where the signal-to-noise ratios 20000 random 50-bp sequences were calculated. The threshold was determined so that 60% of the sequences were discarded (grey area).

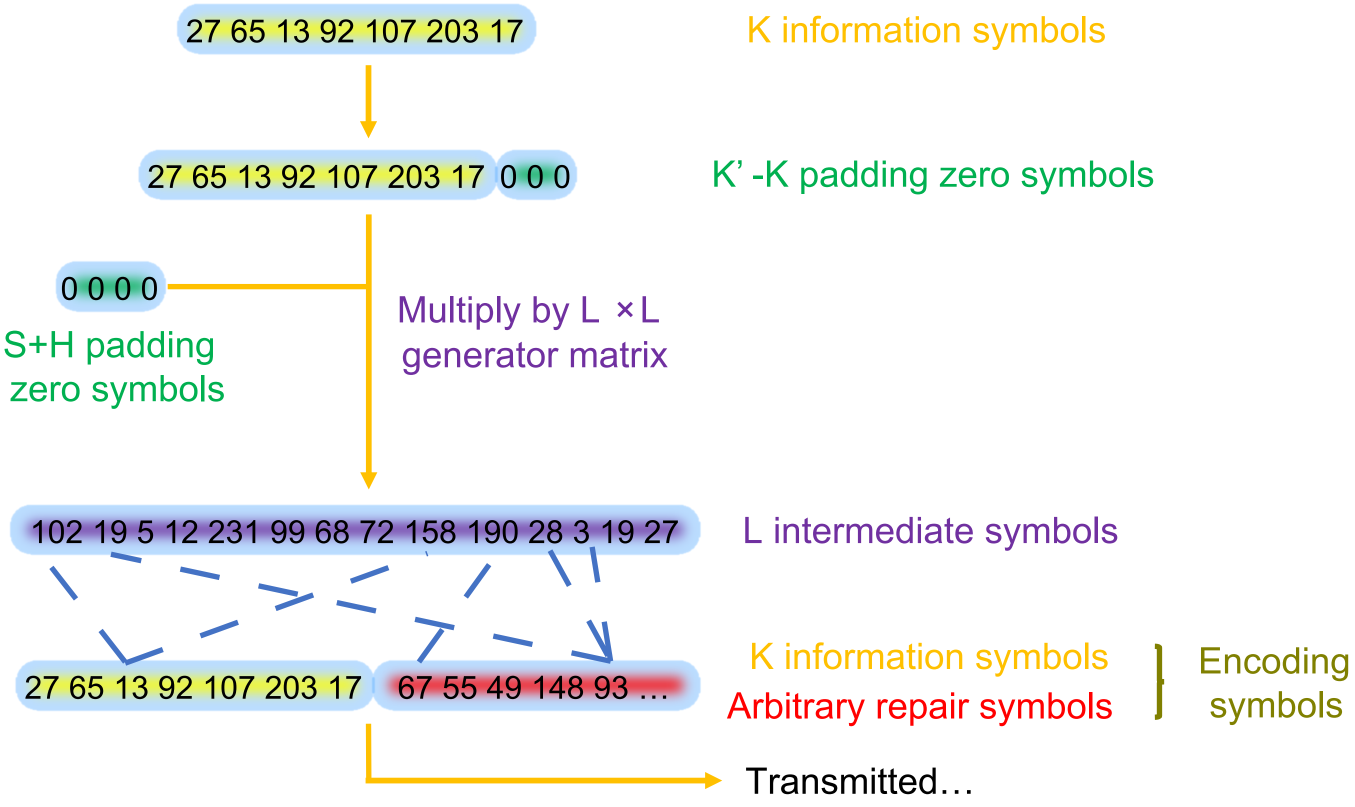

Supplementary Figure 9: Encoding process of RaptorQ code

Information symbols are first padded with certain numbers of 0s, and then multiplied by the generator matrix, generating intermediate symbols. XOR operations are performed on the intermediate symbols according to the designed degree distribution, to simultaneously generate information symbols and arbitrary numbers of repair symbols. The numbers in the figure are for demonstration only, which do not reflect the actual calculations of RaptorQ code.

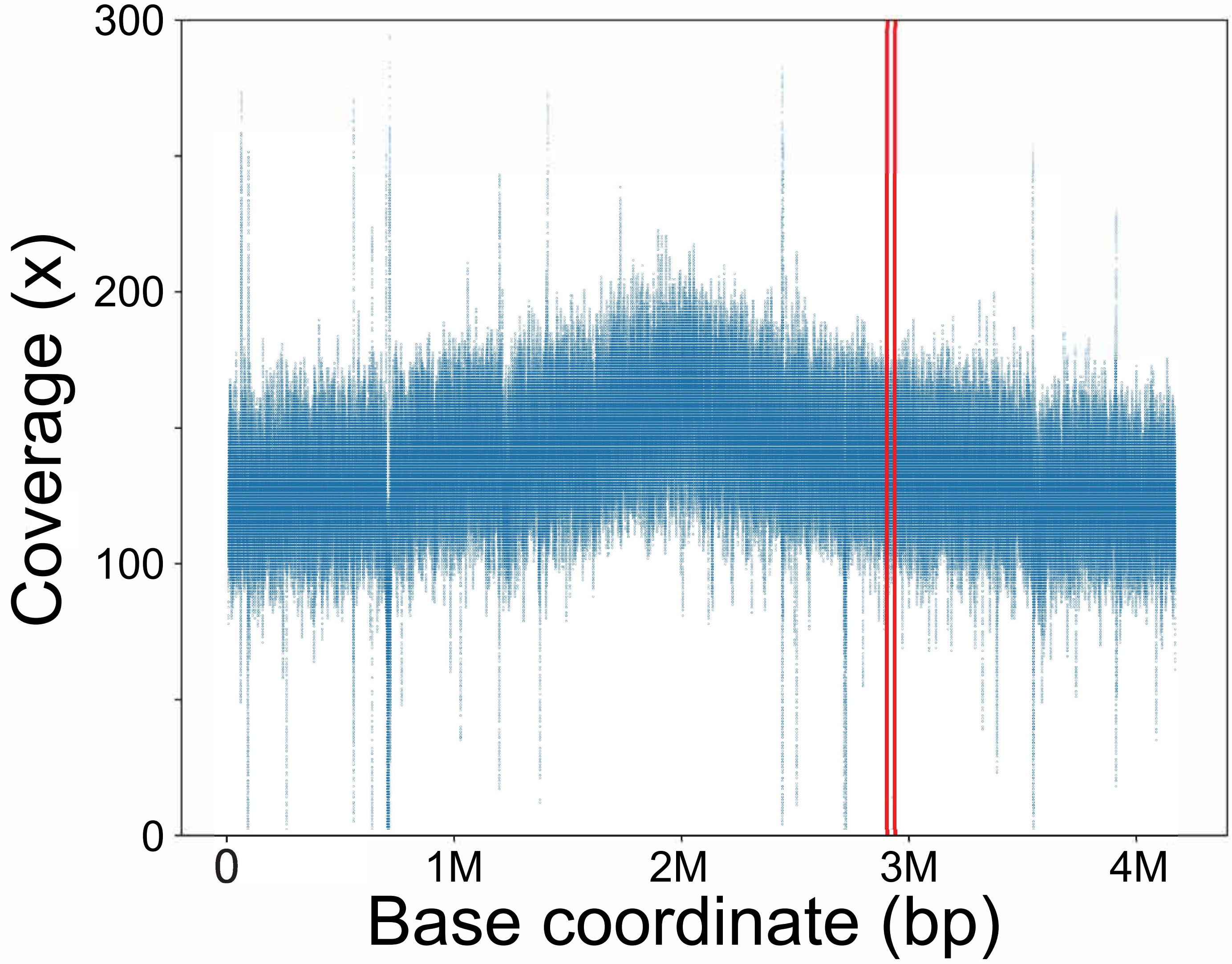

Supplementary Figure 10: Sequencing coverage of bacterial genome in Hi-seq
Here the strain was *H. bluephagenesis* TD01 with information DNA (coded by BCH code) integrated, which was same as the illustration in Fig. 4a. Each dot corresponds to a base. The position of information DNA was 2905575-2940094 bp (labeled with red lines).

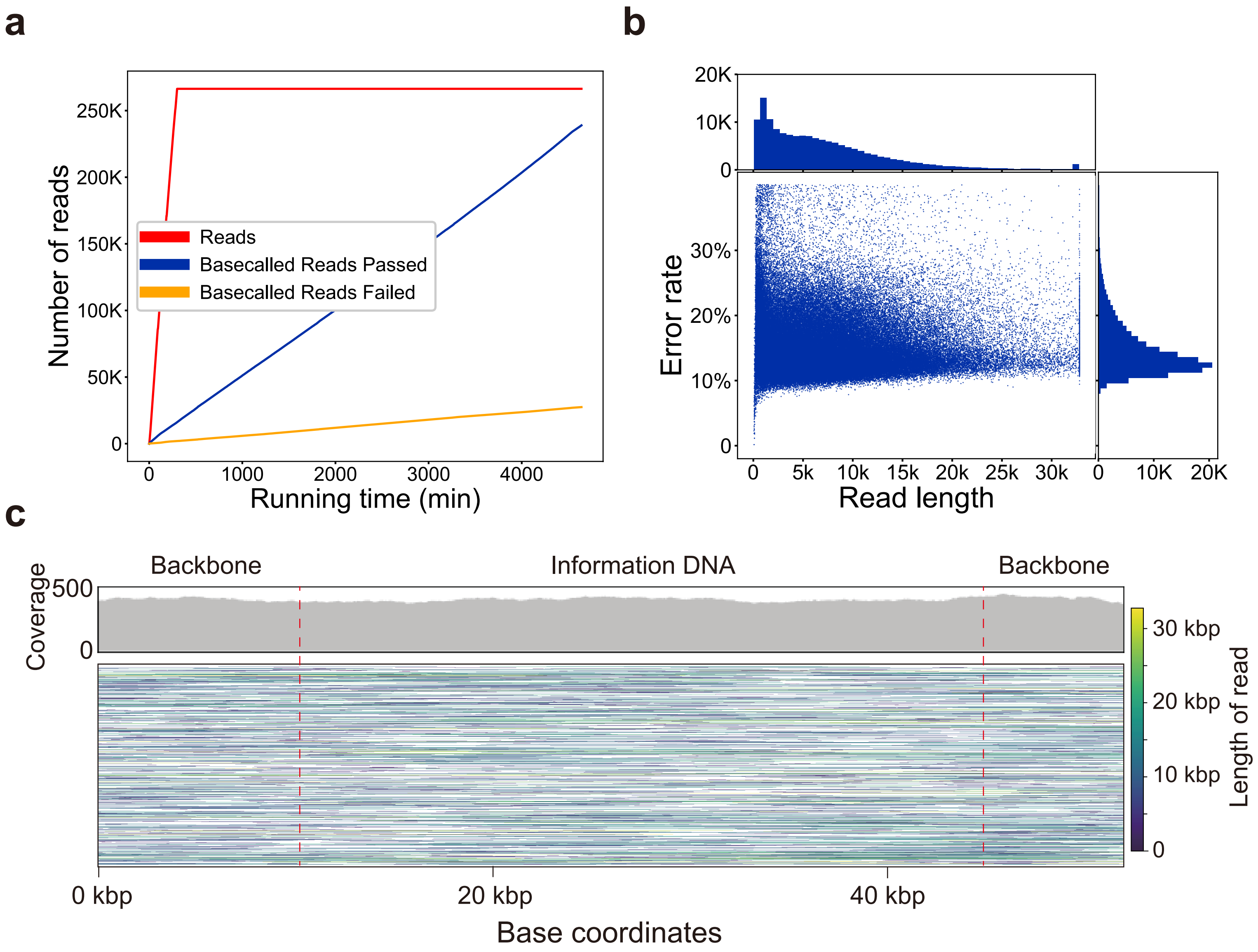

Supplementary Figure 11: Analysis of raw reads in nanopore sequencing
a The number of sequenced reads (in the form of electric signal) and base-called reads (passed/failed) during sequencing process. Base-calling was performed along with sequencing, though with a lower speed. **b** Scatter plot of the length and error rate of the reads. Each point corresponds to a read. The upper histogram shows the distribution of read lengths; the right histogram shows the distribution of error rates. **c** Layout of reads intersecting with information DNA. Red dotted lines represented the boundary of information DNA. Colors indicated the length of the reads. The grey graph above illustrated the coverage.

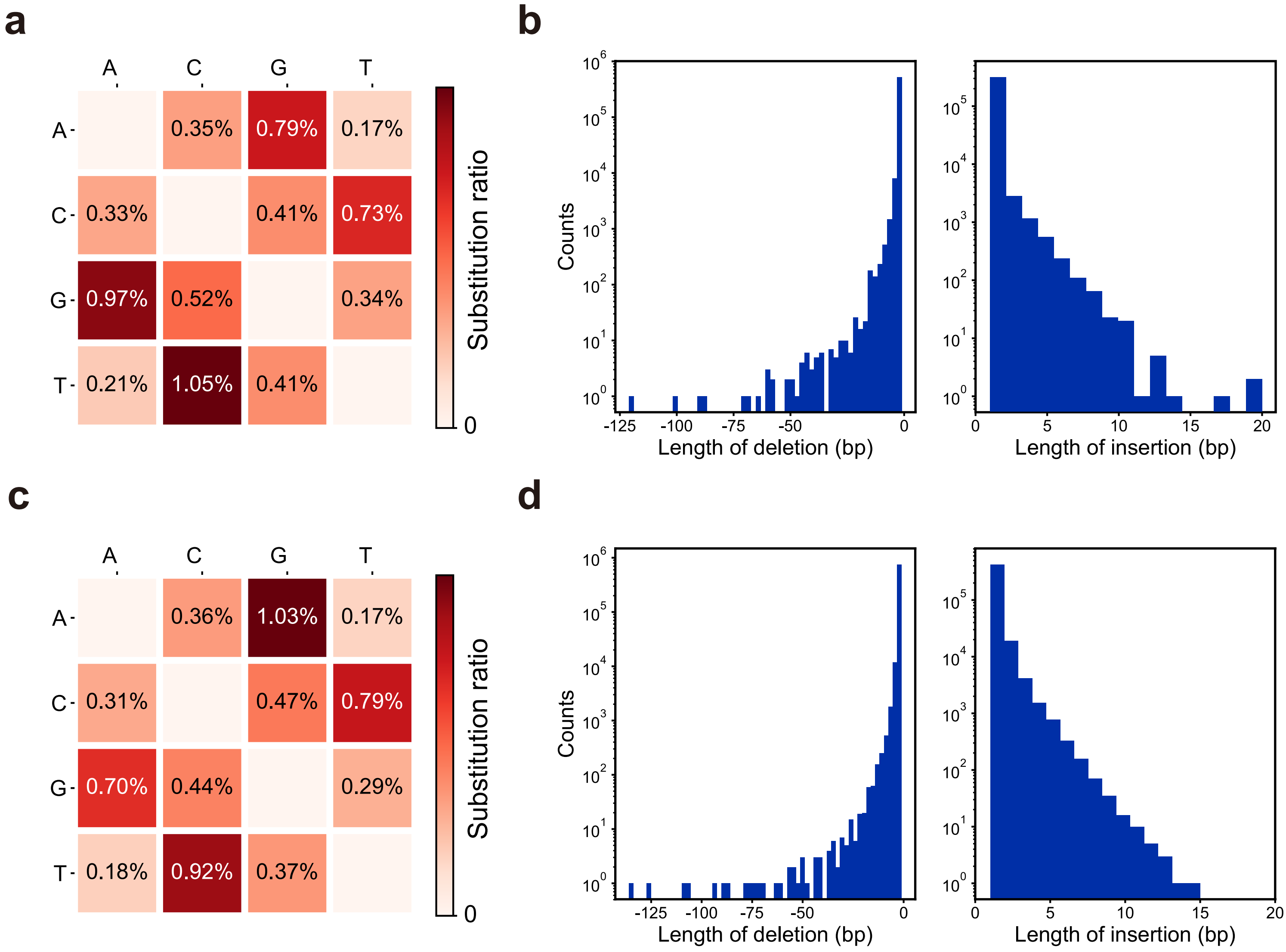

Supplementary Figure 12: Comparison of nanopore sequencing results of BCH-coded information DNA (encoding region) and non-encoding region

The data of the encoding region came from 1,652 reads (13,731,237bp in total); the data of the non-encoding region came from the sequencing results of the opposite position of the encoding region in the genome, which contained 2,898 reads (20,319,898bp in total). **a** Matrix of base substitution frequency of BCH-coded information DNA. **b** Distribution of insertions and deletions of BCH-coded information DNA. **c** Matrix of base substitution frequency of non-encoding region. **d** Distribu-tion of insertions and deletions of non-encoding region.

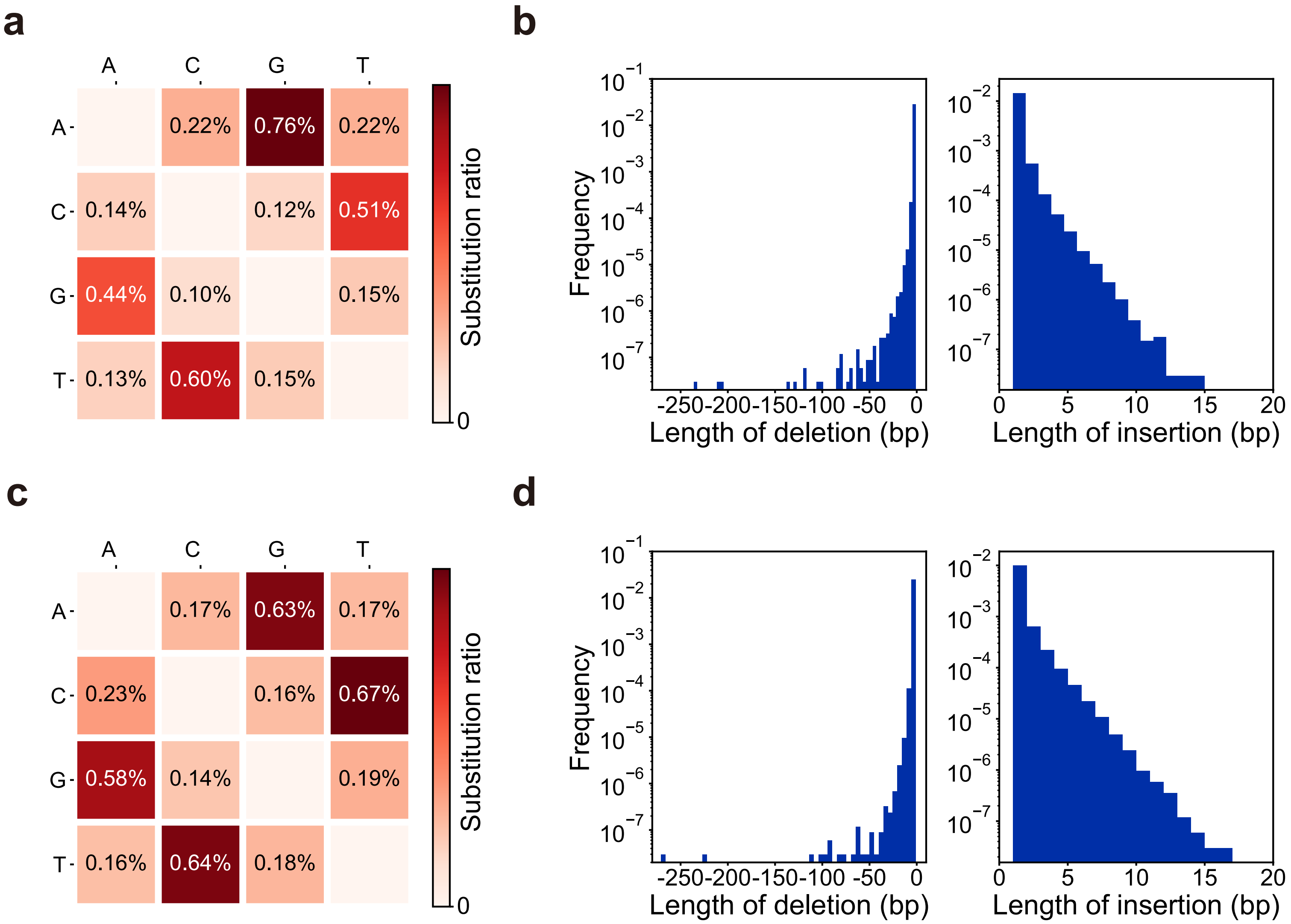

Supplementary Figure 13: Comparison of nanopore sequencing results of MEPCAL-coded information DNA (encoding region) and non-encoding region

The data of the encoding region came from 6,908 reads (33,947,519bp in total); the data of the non-encoding region came from the sequencing results of the relative position of the encoding region in the genome, which contained 3,831 reads (23,653,240bp in total). **a** Matrix of base substitution frequency of MEPCAL-coded information DNA. **b** Distribution of insertions and deletions of MEPCAL-coded information DNA. **c** Matrix of base substitution frequency of non-encoding region. **d** Distribution of insertions and deletions of non-encoding region.

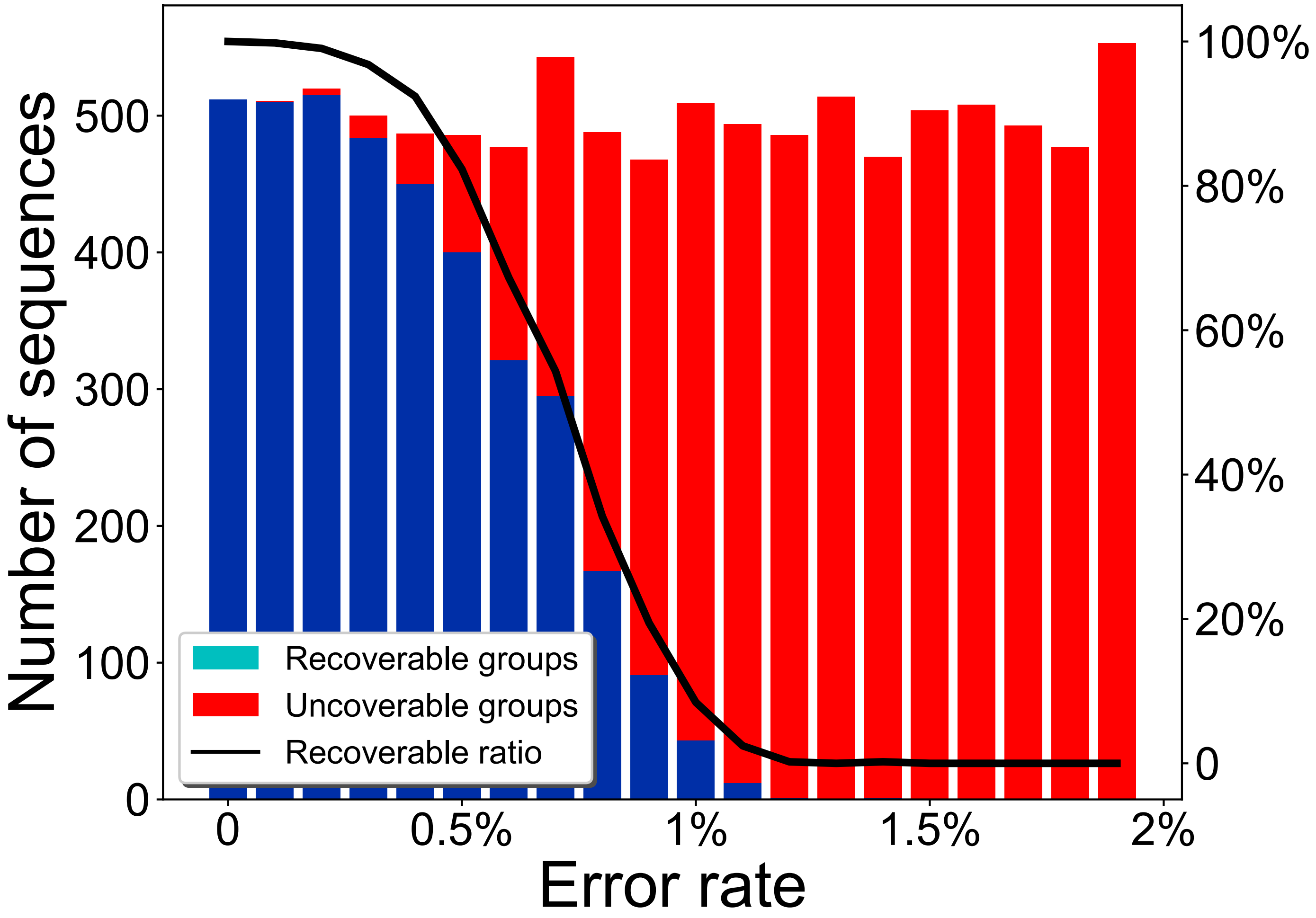

Supplementary Figure 14: Decoding simulation of BCH-coded sequence under different error rates
A total of 10,000 simulations were performed. In each simulation, a random proportion (0-2%) of errors were added to the information DNA, which was then decoded by BCH decoder. Substitutions were exclusively added to the sequences since indels cannot be processed by BCH code.

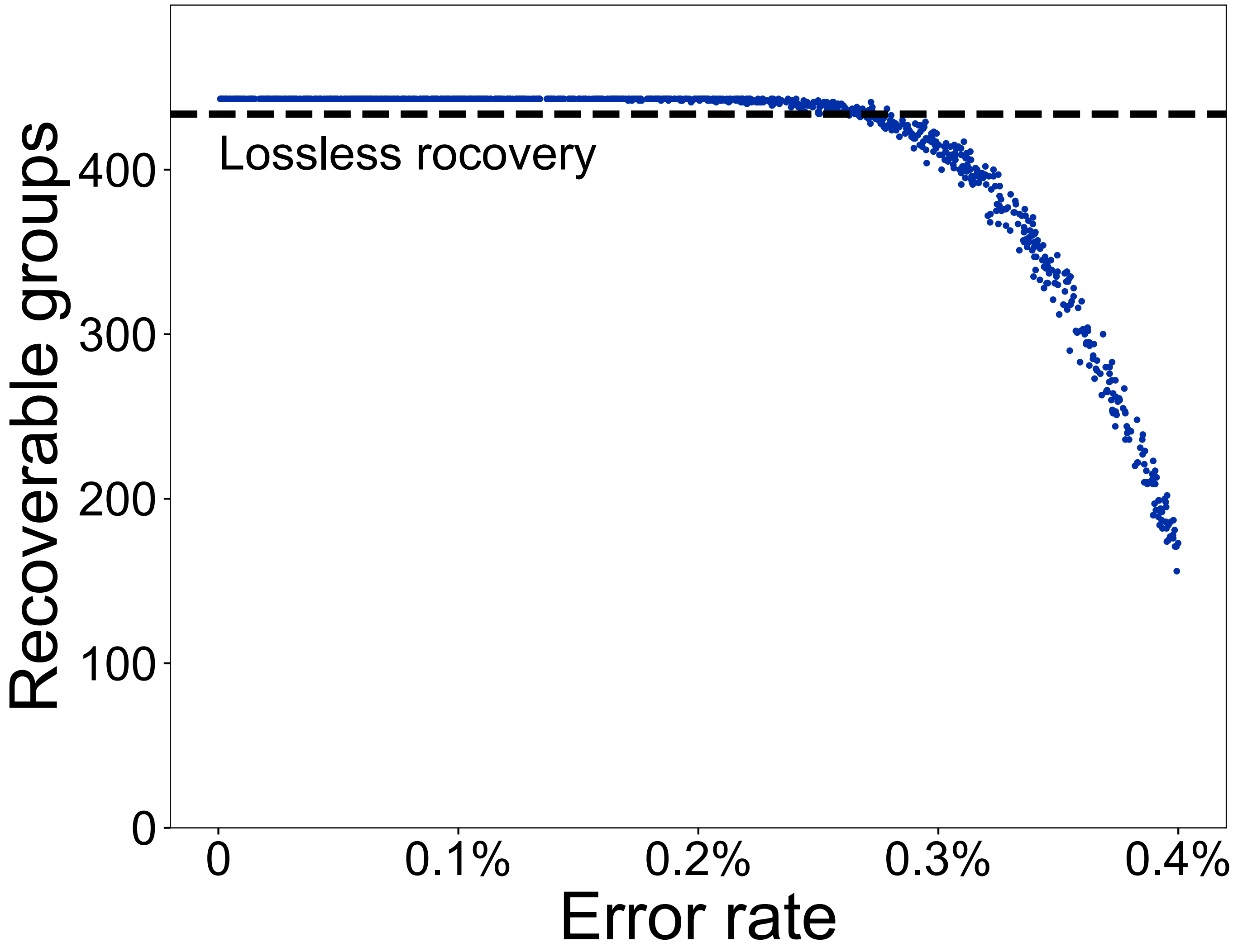

Supplementary Figure 15: Decoding simulation of scale-up storage with MEPCAL under different error rates

A total of 1,000 simulations were performed. In each simulation, a random proportion (0-0.4%) of errors were added to the information DNA, which was then decoded by MEPCAL decoder. Three types of errors (substitutions, insertions, deletions) were equally added. Certain number of encoding groups can be recovered from each simulated sequence. Here the sufficient condition for error-less informa-tion retrieval was recovery of at least 434 (dashed line) out of the 443 encoding groups.

Table S1. Screened integrases and excisionases that can be utilized in *H.bluphagenesis* and corresponding recognition sites. The first 16 rows indicate the integrases corresponding to the 16 attB sites in attB array. These attB sites were orderly connected (from top to bottom in the table) to form the attB array. The attP sites were located on the plasmid for integration. The last 4 rows indicate the excisionases and the corresponding recognition sites. These excisionases can be used for excision of vector sequences after integration of information DNA (as illustrated in Fig. S6).

Integrase	Sequence of attB site (5'→3')	Sequence of attP site (5'→3')
Bxb1[25]	tcggccggcttgtcgacgacggcggtctccg tcgtcaggatcatccgggc	gtcgtggtttgtctggtcaaccaccgcggtct cagtggtgtacggtacaaacccсgac
phiC31[26]	tgcgggtgccagggcgtgcccttgggctccc cgggcgcgtactcc	gtgccccaactggggtaacctttgagttctctc agttggggg
TP901[25]	atgccaacacaattaacatctcaatcaaggta aatgctttttgcttttttgc	taaaaggagtttttagttaccttaattgaaataa acgaaataaaaactcgcaat
Int2[1]	ggacggcgcagaaggggagtagctcttcgc cggaccgtcgacatactgctcagctcgtc	gctcatgtatgtgtctacgcgagattctcgccc gagaacttctgcaaggcactgctcttggct
Int3[1]	gtttgtaaaggagactgataatggcatgtaca actatactcgtcggtaaaaaggcatcttat	atggataaaaaaatacagcgtttttcatgtaca actatactagttgtagtgcctaaataatgctt
Int4[1]	ttccaaagagcgcccaacgcgacctgaaatt tgaataagactgctgcttgtgtaaaggcgatg att	caaaaattacaaagttttcaacccttgatttgaa ttagcggtcaaataatttgtaattcgttt
Int5[1]	gagcgccggatcagggagtggacggcctg ggagcgctacacgctgtggctgcggtcggt gc	ccctaatacgcaagtcgataactctcctggga gcgttgacaacttgcgcaccctgatctg
Int7[1]	agacgagaaacgttccgtccgtctgggtcag ttgggcaaagttgatgaccgggtcgtccgtt	gtgttataaacctgtgtgagagttaagtttacat gcctaaccttaacttttacgcaggttcagctt
Int8[1]	caatcatcagataactatggcggcacgtgcat taaccacggttgtatcccgtctaaagtactcgt	ttaataaactatggaagtatgtacagtcttgca atgttgagtgaacaaacttccataataaaat
Int11[1]	atggattttgcagattcccagatgcccctaca gaaagaggtacaaaacatttattggaattaatt	gtttatatgtttactaataagacgctctcaaccc ataaagtcttattagtaaacatatttcaact
Int12[1]	gttcgtggtaactatgggtggtacaggtgcca cattagttgtaccatttatgtttatgtggttaac	tttttgtatgttagttgtgtcactgggtagaccta aatagtgacacaactgctattaaaatttaa
Int13[1]	cgcatacattgttgttgtttttccagatccagttg gtcctgtaaatataagcaatccatgtgagt	caataacggttgtatttgtagaacttgaccagtt gttttagtaacataaatacaactccgaata
Bxb1[25]	tcggccggcttgtcgacgacggcggtctccg tcgtcaggatcatccgggc	gtcgtggtttgtctggtcaaccaccgcggtct cagtggtgtacggtacaaaccccgac
TP901[25]	atgccaacacaattaacatctcaatcaaggta aatgctttttgcttttttgc	taaaaggagtttttagttaccttaattgaaataa acgaaataaaaactcgcaat

PhiC31[26]	tgcgggtgccagggcgtgcccttgggctcccgggcgcgtactcc	gtgccccaactggggtaacctttgagttctctcagttgggggg
HibF[2]	aatacaagacaattggggccaaactgtccatatcat	caaaagatgaaacatttggggccattttgactcatagag
Flp[28,29]	gaagttcctattccgaagttcctattctctagaaagtataggaacttc	
Dre[30]	taactttaaataatgccaattatttaaagtta	
Scre[31]	ctcgtgtccgataactgtaattatcggacacgag	
Vcre[31]	tcaatttctgagaactgtcattctcggaaattga	

Table S2. Composition of plasmids used in this study.

Strain	Plasmid vector	Integrase gene	Resistance gene	DNA fragments carried(1st)	DNA fragments carried(2nd)
H. bluephagenesis TD01	5	Integrase 5	C^+	ST1-ST6	FT1-FT2
	7	Integrase 7	K^+/ SPC^{+*}	/	FT3-FT4
	12	Integrase 12	Tet^+	ST7-ST10	FT5-FT6
E. coli TOP10	5	Integrase 5	C^+	ST1-ST6	/
	12	Integrase 12	K^+	ST7-ST10	/

* The gene of K^+/ SPC^+ were expressed in *E. coli* and *H. bluephagenesis* respectively.

Table S3. Sequences of DNA elements (except attB sites and attP sites) used in this work. These elements constituted the plasmids for integration together with the attP sites, which carried the DNA sequence encoding information. For the specific composition of these elements in the plasmid, please refer to the genome map in online materials.

Element	Description	Sequence (5’→3’)
oriT	Origin of replication	cttttccgctgcataaccctgcttcggggtcattatagcgattttttcggtatatccatcctttttcgcacgatatacaggattttgccaaagggttcgtgtagactttccttggtgtatccaacggcgtcagccgggcaggataggtgaagtaggcccacccgcgagcgggtgttccttcttcactgtcccttattcgcacctggcggtgctcaacgggaatcctgctctgcgaggctggccgta
oriR6k	Origin of replication	Caagatccgcagttcaacctgttgatagtacgtactaagctctcatgtttcacgtactaagctctcatgtttaacgtactaagctctcatgtttaacgaactaaaccctcatggctaacgtactaagctctcatggctaacgtactaagctctcatgtttcacgtactaagctctcatgtttgaacaataaaattaatataaatcagcaacttaaatagcctctaaggttttaagttttataagaaaaaaaagaatatataaggcttttaaagcttttaaggtttaacggttgtggacaacaagccagggatgtaacgcactgagaagcccttagagcctctcaaagcaattttgagtgacacaggaacacttaacggctgacatgg
Chl^{R}	Chloramphenicol resistance gene	atggagaaaaaaatcactggatataccaccgttgatatatcccaatggcatcgtaaagaacattttgaggcatttcagtcagttgctcaatgtacctataaccagaccgttcagctggatattacggcctttttaaagaccgtaaagaaaaataagcacaagttttatccggcctttattcacattcttgcccgcctgatgaatgctcatccggaatttcgtatggcaatgaaagacggtgagctggtgatatgggatagtgttcacccttgttacaccgttttccatgagcaaactgaaacgttttcatcgctctggagtgaataccacgacgatttccggcagtttctacacatatattcgcaagatgtggcgtgttacggtgaaaacctggcctatttccctaaagggtttattgagaatatgtttttcgtctcagccaatccctgggtgagtttcaccagttttgatttaaacgtggccaatatggacaacttcttcgcccccgttttcaccatgggcaaatattatacgcaaggcgacaaggtgctgatgccgctggcgattcaggttcatcatgccgtttgtgatggcttccatgtcggcagaatgcttaatgaattacaacagtactgcgatgagtggcagggcggggcgtaa
Chl^{R}-P	Promotor of Chloramphenicol resistance gene	tgatcggcacgtaagaggttccaactttcaccataatgaaataagatcactaccgggcgtatttttgagttatcgagattttcaggagctaaggaagctaaa
Kan^{R}	Kanamycin resistance gene	atgagccatattcaacgggaaacgtcttgctcgaggccgcgattaaattccaacatggatgctgatttatatgggtataaatgggctcgcgataatgtcgggcaatcaggtgcgacaatctatcgattgtatgggaagcccgatgcgccagagttgtttctgaaacatggcaaaggtagcgttgccaatgatgttacagatgagatggtcagactaaactggctgacggaatttatgcctcttccgaccatcaagcattttatccgtactcctgatgatgcatggttactcaccactgcgatccccgggaaaaca

		gcattccaggtattagaagaatatcctgattcaggtgaaaatattgttgatgcgctggcagtgtt<br>cctgcgccggttgcattcgattcctgtttgtaattgtccttttaacagcgatcgcgtatttcgtctc<br>gctcaggcgcaatcacgaatgaataacggtttggttgatgcgagtgattttgatgacgagcgt<br>aatggctggcctgttgaacaagtctggaaagaaatgcataagcttttgccattctcaccggatt<br>cagtcgtcactcatggtgatttctcacttgataaccttatttttgacgaggggaaattaataggtt<br>gtattgatgttggacgagtcggaatcgcagaccgataccaggatcttgccatcctatggaact<br>gcctcggtgagttttctccttcattacagaaacggctttttcaaaaatatggtattgataatcctg<br>atatgaataaattgcagtttcatttgatgctcgatgagtttttctaa
Spc^R	Spectinomycin resistance gene	atgcgctcacgcaactggtccagaaccttgaccgaacgcagcggtggtaacggcgcagtg<br>gcggttttcatggcttgttatgactgtttttttggggtacagtctatgcctcgggcatccaagca<br>gcaagcgcgttacgccgtgggtcgatgtttgatgttatggagcagcaacgatgttacgcagc<br>agggcagtcgccctaaaacaaagttaaacatcatgagggaagcggtgatcgccgaagtat<br>cgactcaactatcagaggtagttggcgtcatcgagcgccatctcgaaccgacgttgctggcc<br>gtacatttgtacggctccgcagtggatggcggcctgaagccacacagtgatattgatttgctg<br>gttacggtgaccgtaaggcttgatgaaacaacgcggcgagctttgatcaacgaccttttgga<br>aacttcggcttcccctggagagagcgagattctccgcgctgtagaagtcaccattgttgtgca<br>cgacgacatcattccgtggcgttatccagctaagcgcgaactgcaatttggagaatggcagc<br>gcaatgacattcttgcaggtatcttcgagccagccacgatcgacattgatctggctatcttgct<br>gacaaaagcaagagaacatagcgttgccttggtaggtccagcggcggaggaactctttgat<br>ccggttcctgaacaggatctatttgaggcgctaaatgaaaccttaacgctatggaactcgccg<br>cccgactgggctggcgatgagcgaaatgtagtgcttacgttgtcccgcatttggtacagcgc<br>agtaaccggcaaaatcgcgccgaaggatgtcgctgccgactgggcaatggagcgcctgc<br>cggcccagtatcagcccgtcatacttgaagctagacaggcttatcttggacaagaagaagat<br>cgcttggcctcgcgcgcagatcagttggaagaatttgtccactacgtgaaaggcgagatca<br>ccaaggtagtcggcaaataa
Tet^R	Tetracycline resistance gene	atgaaatctaacaatgcgctcatcgtcatcctcggcaccgtcaccctggatgctgtaggcata<br>ggcttggttatgccggtactgccgggcctcttgcgggatatcgtccattccgacagcatcgc<br>cagtcactatggcgtgctgctagcgctatatgcgttgatgcaatttctatgcgcacccgttctc<br>ggagcactgtccgaccgctttggccgccgcccagtcctgctcgcttcgctacttggagccac<br>tatcgactacgcgatcatggcgaccacacccgtcctgtggatcctctacgccggacgcatc<br>gtggccggcatcaccggcgccacaggtgcggttgctggcgcctatatcgccgacatcacc<br>gatggggaagatcgggctcgccacttcgggctcatgagcgcttgtttcggcgtgggtatggt<br>ggcaggccccgtggccgggggactgttgggcgccatctccttgcatgcaccattccttgcg<br>gcggcggtgctcaacggcctcaacctactactgggctgcttcctaatgcaggagtcgcataa<br>gggagagcgtcgaccgatgcccttgagagccttcaacccagtcagctccttccggtgggc<br>gcggggcatgactatcgtcgccgcacttatgactgtcttctttatcatgcaactcgtaggacag<br>gtgccggcagcgctctgggtcattttcggcgaggaccgctttcgctggagcgcgacgatga<br>tcggcctgtcgcttgcggtattcggaatcttgcacgccctcgctcaagccttcgtcactggtcc

		cgccaccaaacgtttcggcgagaagcaggccattatcgccggcatggcggccgacgcgc<br>tgggctacgtcttgctggcgttcgcgacgcgaggctggatggccttccccattatgattcttct<br>cgcttccggcggcatcgggatgcccgcgttgcaggccatgctgtccaggcaggtagatga<br>cgaccatcagggacagcttcaaggatcgctcgcggctcttaccagcctaacttcgatcactg<br>gaccgctgatcgtcacggcgatttatgccgcctcggcgagcacatggaacgggttggcatg<br>gattgtaggcgccgccctataccttgtctgcctccccgcgttgcgtcgcggtgcatggagcc<br>gggccacctcgacctga
pInt5	Integrase5 gene	atgcctggtatgaccaccgaaaccggtccggatcctgcaggtctgattgacctgttttgtcgt<br>aaaagcaaagcagttaaaagccgtgcaaatggtgcaggtcagcgtcgtaaacaagaaatta<br>gcattgcagcacaagaaaccctgggtcgtaaagttgcagcactgctgggtatgcaggttcgt<br>catgtttggaaagaagttggtagcgcaagccgttttcgtaaaggtaaagcacgtgatgatca<br>gagcaaagcactgaaagccctggaaagcggtgaagttggtgcactgtggtgttatcgtctg<br>gatcgttgggatcgtggtggtgccggtgcaattctgaaaattatcgaaccggaagatggtat<br>gcctcgtcgtctgctgtttggttgggatgaagataccggtcgtccggttctggatagcaccaa<br>taaacgtgatcgcggtgaactgattcgtcgtgcagaagaagcacgcgaagaagcagaaaa<br>actgagcgaacgtgttcgtgataccaaagcacatcagcgtgaaaatggtgaatgggttaatg<br>cccgtgcaccgtatggtctgcgtgttgttctggttaccgttagtgatgaagagggtgatgaata<br>tgatgaacgtaaactggcagcagatgatgaagatgcgggtggtcctgatggtctgaccaaa<br>gcagaagcagcccgtctggtttttaccctgccggttaccgatcgtctgagctatgcaggcac<br>cgcacatgcaatgaatacccgtgaaattccgagcccgaccggtggtccgtggattgcagtta<br>ccgtgcgtgatatgattcagaatccggcatatgcgggttggcagaccacaggtcgtcaggat<br>ggtaaacagcgtcgtctgaccttttataacggtgaaggtaaacgtgttagcgttatgcatggtc<br>ctccgctggtgaccgatgaagaacaagaagccgcaaaagcagccgttaaaggtgaagatg<br>gtgttggtgttccgctggatggtagcgatcatgatacccgtcgcaaacatctgctgagcggtc<br>gtatgcgttgtccgggttgtggtggtagctgtagctatagcggtaatggttatcgttgttggcgt<br>agcagtgtgaaaggtggttgtccggcaccgacctatgttgcacgtaaaagcgttgaagaata<br>tgttgcatttcgttgggcagcaaaattagcagcaagcgaaccggatgatccgtttgttattgca<br>gttgcagatcgctgggcagcactgacccatccgcaggcaagcgaagatgaaaagtatgca<br>aaagccgcagttcgtgaagccgaaaaaaatctgggtcgcctgctgcgtgatcgtcagaatg<br>gtgtttatgatggtccggcagaacagtttttgcccctgcatatcaagaagcactgagcaccct<br>gcaggcagccaaagatgcagttagcgaaagcagcgcaagcgcagcagttgatgttagctg<br>gattgttgatagcagcgattatgaagaactgtggctgcgtgcaaccccgaccatgcgtaatg<br>caattattgatacctgcatcgatgaaatttgggttgcaaaaggccagcgtggtcgtccgtttga<br>tggtgatgaacgcgttaaaatcaaatgggcagcccgtacctaa
pInt7	Integrase7 gene	atgaaagtggccatttatgttcgtgttagcaccgatgaacaggccaaagaaggttttagcattc<br>cggcacagcgtgaacgtctgcgtgcattttgtgcaagccagggttgggaaattgtgcaaga<br>atatattgaagaaggttggagcgcaaaagatctggatcgtccgcagatgcagcgtctgctga<br>aagatatcaaaaaaggcaacattgatattgtgctggtgtatcgtctggatcgcctgacccgta

		gcgttctggatctgtatctgctgctgcagacctttgaaaaatacaatgtggcatttcgtagcgc caccgaagtttatgataccagcaccgcaatgggtcgtctgtttattaccctggttgcagcactg gcacagtgggaacgtgaaaatctggcagaacgtgttaaatttggtatcgagcagatgatcga tgaaggtaaaaaaccgggtggtcatagcccgtatggttacaaatttgataaagacttcaattg caccattattgaggaagaagcagacgttgttcgtatgatctatcgcatgtattgtgatggttatg gctatcgtagcattgcagatcgtctgaatgaactgatggttaaaccgcgtattgccaaagaat ggaatcataatagcgtgcgtgatatcctgaccaacgatatctatattggcacctatcgttgggg tgataaagttgttccgaataatcatccgcctattattagcgaaaccctgttcaaaaaagcccag aaagaaaaagaaaaacgtggcgttgatcgtaaacgcgttggtaaatttctgtttaccggtctg ctgcagtgtggtaattgtggtggccataaaatgcagggccattttgataaacgtgagcagaa aacctattaccgttgtaccaaatgtcaccgcattaccaacgaaaaaaacattctggaaccgct gctggatgaaattcagctgctgattaccagcaaagaatactttatgagcaaattcagcgaccg ctatgatcagcaagaggttgttgatgttagcgcactgacaaaagaactggaaaaaatcaaac gccagaaagagaaatggtacgatctgtatatggatgatcgtaacccgattccgaaagaaga actgtttgccaaaattaacgaactgaacaaaaaagaagaagaaatctatagcaagctgagc gaagtggaagaagataaagaaccggttgaagagaaatataaccgcctgagcaaaatgatc gattttaaacagcagtttgagcaggccaacgactttaccaaaaaagagctgctgttcagcatc ttcgaaaagattgtgatttatcgcgagaaaggcaagctgaaaaaaatcaccctggattacac cctgaaataa
pInt12	Integrase12 gene	atgaaagtggccatttatacccgtgttagcagcgcagaacaggcaaatgaaggttatagcat tcacgagcagaagaagaaactgatcagctattgcgaaatccacgattggaacgagtataaa gtttttaccgatgcaggtattagcggtggtagcatgaaacgtccggcactgcaaaaactgatg aaacatctgagttcatttgatctggtgctggtgtataaactggatcgtctgacccgtaatgttcg tgatctgctggatatgctggaagaatttgaacagtataacgtgagctttaaaagcgccaccga agtttttgataccaccagtgcaattggcaaactgtttattaccatggttggtgcaatggcagaat gggaacgtgaaaccattcgtgaacgtagcctgtttggtagccgtgcagcagttcgtgaaggt aactatattcgtgaagcaccgttttgctatgataacattgaaggtaaactgcacccgaacgaat atgccaaagttattgatctgattgtgagcatgttcaaaaaaggcattagcgccaatgaaattgc acgtcgtctgaatagcagcaaagttcatgttccgaacaaaaaaagctggaatcgtaatagcc tgattcgtctgatgcgtagtccggttctgcgtggtcataccaaatatggtgatatgctgattgaa aacacccatgaaccggtgctgagcgaacatgattataatgcaattaacaacgccatcagca gcaaaacccataaaagcaaagttaaacaccatgccatttttcgtggtgcactggtttgtccgc agtgtaatcgtcgtctgcatctgtatgcaggcaccgttaaagatcgtaaaggctataaatacg atgtgcgtcgctataaatgtgaaacctgcagcaaaaacaaagatgtgaagaatgtgagcttc aacgaaagcgaagtggaaaacaaattcgtcaatctgctgaaaagctacgagctgaacaaat ttcatatccgtaaagtggaaccggtgaaaaaaatcgagtatgacatcgataagattaacaaac agaaaattaactatacccgcagttggagcctgggctatattgaagatgatgaatatttcgagct gatggaagaaatcaacgccaccaaaaaaatgatcgaagaacagaccaccgagaataaac

		agagcgttagcaaagagcagattcagagcattaacaactttatcctgaaaggctgggaagaactgaccatcaaagataaagaggaactgattctgagcaccgtggataaaatcgaatttaacttcatcccgaaagataaaaaacataaaaccaataccctggatattaacaatattcactttaaattctaa
RiboJ	Ribozyme gene	Agctgtcaccggatgtgctttccggtctgatgagtccgtgaggacgaaacagcctctacaaataattttgtttaa
B0015^{T}	B0015 dblTerm terminator	Ccaggcatcaaataaaacgaaaggctcagtcgaaagactgggcctttcgttttatctgttgtttgtcggtgaacgctctctactagagtcacactggctcaccttcgggtgggcctttctgcgtttata
T7Tphi^{T}	T7Tphi terminator	Ctgctaacaaagcccgaaaggaagctgagttggctgctgccaccgctgagcaataactagcataaccccttggggcctctaaacgggtcttgaggggttttttgctgaaaggaggaactatatccggat
dbT7TE^{T}	dbT7TE terminator	gtcatgcttgccatctgttttcttgcaagat

Table S4. Primers used to verify BCH-coded information DNA (ST1-10) in passaging experiments.

Primer	Sequence (5'→3')	Covered region of the PCR reaction
TENSEQ1_FW	GCTTTTAAGGTTTAACGGTTGTGG	The junction of ST1 and the genome
TENSEQ1_RV	AACTCCATTAAGTTAACGTGCAGC	
TENSEQ2_FW	CCTATCCGGCATTTCTCATCTTTCC	The junction of ST6 and the genome; The junction of ST7 and the genome
TENSEQ2_RV	TCAGACCAGAAATGCCGGATCTT	
TENSEQ3_FW	CCTTGTCCCAGAAACTGCAACA	The junction of ST10 and the genome
TENSEQ3_RV	CATCCACCTTTACCCATTAACTCC	

Table S5. Primers covering the whole BCH-coded information DNA. These primers were used to detect mutations during passaging experiments. "FW" primer and "RV" primer with the same serial number were used in one PCR reaction. For the specific positions of these primers in the sequence, please refer to the genome map in online materials.

Primer	Sequence (5'→3')
TENSEQ1_FW	GCTTTTAAGGTTTAACGGTTGTGG
TENSEQ1_RV	AACTCCATTAAGTTAACGTGCAGC
TENSEQ2_FW	CCTATCCGGCATTTCTCATCTTTCC
TENSEQ2_RV	TCAGACCAGAAATGCCGGATCTT
TENSEQ3_FW	CCTTGTCCCAGAAACTGCAACA
TENSEQ3_RV	CATCCACCTTTACCCATTAACTCC
TENSEQ4_FW	GGTAACTACCCTGATTCGGATACG
TENSEQ4_RV	TGCAACACATGATGAGACCTATACG
TENSEQ5_FW	AGGGTCCACTCACAGGAATAGTGA
TENSEQ5_RV	CTGCCTCTCGTTACCATGTTGGA
TENSEQ6_FW	ATCCCAGCTAAGTGTGAGGTCA
TENSEQ6_RV	CGTACGCTATAGGTCAGACCCA
TENSEQ7_FW	ATGCGCTGTACCGACTCACTTA
TENSEQ7_RV	CAACACAGTTTATGCCTCACACAT
TENSEQ8_FW	AGGAAAGAGGTACGGCATTAGC
TENSEQ8_RV	GCCTTGGCCATACGAGTATTACT
TENSEQ9_FW	TCAATGCGGCAGAGTTCACTATC
TENSEQ9_RV	GTAAGAGAAAACTCCTCCGTACCTC
TENSEQ10_FW	ATGTTACCGAGCTGAAACACGG
TENSEQ10_RV	GGAACGTGCATACCACCAATTAT
TENSEQ11_FW	CCTGGGTTAACTGAGGAAATTGGA
TENSEQ11_RV	GGCGCTTTTAAAGCTCACGTTAT
TENSEQ12_FW	GCTTTTAAGGTTTAACGGTTGTGG
TENSEQ12_RV	TGCCGTAACGATTGAGTTGTAATCT
TENSEQ13_FW	AAGCCTATACAAGGTACGGCAC
TENSEQ13_RV	GGTTAGCTCATCTAGCATACGCG
TENSEQ14_FW	GCGGAGCTTTAATTGAAAGAGGTG
TENSEQ14_RV	ACTTAAAGACATGTCCTGGATACGC
TENSEQ15_FW	TCTCGTAGGCCTCTTCTGTTTAGC
TENSEQ15_RV	CCGTGTAGCAGAGGCTGTAATT
TENSEQ16_FW	TGTCTCATGCCTTGCGGAACTC

TENSEQ16_RV	GGTAAAGGCTCGGTTCCTTCAATA
TENSEQ17_FW	AAGCAATTGCGTTACCATCCG
TENSEQ17_RV	GGAACAATTTTGGCTGCAGGTATAT
TENSEQ18_FW	ATATAGGTTGCGCTGCCTTGAG
TENSEQ18_RV	GCGCGATACACTCTATATCACCAT

Table S6. Sanger sequencing primers used for MEPCAL-coded information DNA (FT1-6). These primers covered not all the integrated sequence, but the connection site between the integrated sequence and the genome. For the specific positions of these primers in the sequence, please refer to the genome map in online materials.

Primer	Sequence (5’→3’)	Covered region of the PCR reaction
TD01L_FW	CCTCAATAATCAGTACGTTTCGTGT	The junction of FT1 and the genome
LF3_FW	AGTGCTTTGCTCTGATCATCACG	
LF3_RV	CTATACTAACCGATGGCACCGT	The junction of FT2 and FT3
Int7_FW	TCAGACCAGAAATGCCGGATCTT	
LF9_FW	CCTTGTCCCAGAAACTGCAACA	The junction of FT4 and FT5
TENSEQ11_RV	CATCCACCTTTACCCATTAACTCC	
LF6_FW	CAAGCGAGCCCAATGGATGTTA	The junction of FT6 and the genome
Sin5R_RV	GACATGAGTTTACGGCGTGTAGT	
LF2_FW	ATTGACGCAATGTCGAGTTCATT	The junction of FT1 and FT2
LF2_RV	ATGTGGACGTTCTAGTGGACCT	
LF8_FW	ATCCGATGTCTGTTGATCCCATTC	The junction of FT3 and FT4
LF8_RV	CCCATATGATCCAATGGCATTCG	
LF5_FW	AACTCCAGATGACCACAATCCAG	The junction of FT5 and FT6
LF5_RV	CCTTTGTCTGGAGTCGAGTCAAAT	

Table S7. Generation time of strains cultured in shaker. For strains with information DNA integrated, antibiotics were added into the culture media.

Strain	Information DNA integrated	Generation time (min)
E. coli TOP10	/	47.26±1.25
E. coli TOP10	ST1-10	44.79±1.11
Halomonas bluephagenesis TD01	/	45.97±0.82
Halomonas bluephagenesis TD01	ST1-10	45.43±0.81

Table S8. Sanger sequencing results of passaging experiments. For the original Sanger sequencing results, please refer to the sequencing results in online materials.

Generation	Number of substitutions	Number of insertions	Number of deletions	Number of total errors
0th	0	0	0	0
100th	0	0	0	0
200th	1^{*}	0	0	1
400th	1	0	0	1
800th	0	0	0	0
1200th	0	0	0	0
2000th	0	0	0	0

*This substitution seemed to be due to sequencing error rather than sequence error, since the position was found to be correct since 800th generation.

Table S9. Error statistics of high-throughput sequencing results of information DNA coded by BCH code (ST1-10).

Sequencing method	Statistical scope	Number of reads	Number of bases	Number of substitutions	Number of insertions	Number of deletions	Number of errors
Hi-seq	Subsample	25,947	2,559,736bp	14,260bp (0.56%)	663bp (0.026%)		14,923bp (0.58%)
nanopore sequencing	Whole genome	137,254	977,967,436 bp	58,687,033bp (6.00%)	34,595,328bp (3.54%)	40,446,427bp (4.14%)	133,728,788bp (13.67%)
	Encoding region	1,652	13,731,237bp	860,914bp (6.27%)	515,830bp (3.76%)	591,223bp (4.31%)	1,967,967 bp (14.33%)
	Subsample of non-encoding region	2,898	20,319,898bp	1,225,927 bp (6.03%)	730,018bp (3.59%)	848,281bp (4.17%)	2,804,226 bp (13.80%)

Table S10. Results of *in silico* simulation for MEPCAL with different *R*, *S* and *ε*.

S	R	ε	mean of $\bar{r}$	S	R	ε	mean of $\bar{r}$
5	4	0.001	0.9915	20	8	0.02	0.7090
5	4	0.005	0.9577	20	8	0.025	0.6437
5	4	0.01	0.9167	20	8	0.03	0.5806
5	4	0.015	0.8769	20	8	0.035	0.5202
5	4	0.02	0.8385	20	8	0.04	0.4640
5	4	0.025	0.8010	20	8	0.045	0.4106
5	4	0.03	0.7644	20	8	0.05	0.3593
5	4	0.035	0.7280	20	10	0.001	0.9797
5	4	0.04	0.6941	20	10	0.005	0.9028
5	4	0.045	0.6607	20	10	0.01	0.8109
5	4	0.05	0.6277	20	10	0.015	0.7220
5	6	0.001	0.9863	20	10	0.02	0.6384
5	6	0.005	0.9330	20	10	0.025	0.5590
5	6	0.01	0.8690	20	10	0.03	0.4852
5	6	0.015	0.8081	20	10	0.035	0.4146
5	6	0.02	0.7497	20	10	0.04	0.3508
5	6	0.025	0.6936	20	10	0.045	0.2894
5	6	0.03	0.6401	20	10	0.05	0.2338
5	6	0.035	0.5886	20	20	0.001	0.9584
5	6	0.04	0.5398	20	20	0.005	0.8015
5	6	0.045	0.4929	20	20	0.01	0.6206
5	6	0.05	0.4473	20	20	0.015	0.4587
5	8	0.001	0.9798	20	20	0.02	0.3170
5	8	0.005	0.9029	20	20	0.025	0.1923
5	8	0.01	0.8129	20	20	0.03	0.0847
5	8	0.015	0.7288	40	4	0.001	0.9928
5	8	0.02	0.6493	40	4	0.005	0.9624
5	8	0.025	0.5758	40	4	0.01	0.9170
5	8	0.03	0.5060	40	4	0.015	0.8663
5	8	0.035	0.4402	40	4	0.02	0.8118
5	8	0.04	0.3791	40	4	0.025	0.7566
5	8	0.045	0.3208	40	4	0.03	0.7039
5	8	0.05	0.2661	40	4	0.035	0.6487
5	10	0.001	0.9737	40	4	0.04	0.5969
5	10	0.005	0.8760	40	4	0.045	0.5504

5	10	0.01	0.7625	40	4	0.05	0.5065
5	10	0.015	0.6579	40	6	0.001	0.9894
5	10	0.02	0.5626	40	6	0.005	0.9473
5	10	0.025	0.4743	40	6	0.01	0.8936
5	10	0.03	0.3915	40	6	0.015	0.8384
5	10	0.035	0.3151	40	6	0.02	0.7815
5	10	0.04	0.2445	40	6	0.025	0.7243
5	10	0.045	0.1778	40	6	0.03	0.6669
5	10	0.05	0.1177	40	6	0.035	0.6132
5	20	0.001	0.9438	40	6	0.04	0.5594
5	20	0.005	0.7419	40	6	0.045	0.5098
5	20	0.01	0.5279	40	6	0.05	0.4606
5	20	0.015	0.3472	40	8	0.001	0.9847
5	20	0.02	0.1928	40	8	0.005	0.9252
5	20	0.025	0.0602	40	8	0.01	0.8488
10	4	0.001	0.9922	40	8	0.015	0.7737
10	4	0.005	0.9612	40	8	0.02	0.7001
10	4	0.01	0.9238	40	8	0.025	0.6291
10	4	0.015	0.8867	40	8	0.03	0.5624
10	4	0.02	0.8505	40	8	0.035	0.4972
10	4	0.025	0.8146	40	8	0.04	0.4388
10	4	0.03	0.7799	40	8	0.045	0.3837
10	4	0.035	0.7452	40	8	0.05	0.3301
10	4	0.04	0.7116	40	10	0.001	0.9810
10	4	0.045	0.6790	40	10	0.005	0.9068
10	4	0.05	0.6467	40	10	0.01	0.8120
10	6	0.001	0.9876	40	10	0.015	0.7199
10	6	0.005	0.9396	40	10	0.02	0.6308
10	6	0.01	0.8817	40	10	0.025	0.5500
10	6	0.015	0.8265	40	10	0.03	0.4741
10	6	0.02	0.7732	40	10	0.035	0.4031
10	6	0.025	0.7223	40	10	0.04	0.3406
10	6	0.03	0.6730	40	10	0.045	0.2809
10	6	0.035	0.6259	40	10	0.05	0.2244
10	6	0.04	0.5806	40	20	0.001	0.9612
10	6	0.045	0.5360	40	20	0.005	0.8054
10	6	0.05	0.4941	40	20	0.01	0.6180
10	8	0.001	0.9821	40	20	0.015	0.4513

10	8	0.005	0.9148	40	20	0.02	0.3110
10	8	0.01	0.8341	40	20	0.025	0.1899
10	8	0.015	0.7586	40	20	0.03	0.0849
10	8	0.02	0.6866	100	4	0.001	0.9929
10	8	0.025	0.6186	100	4	0.005	0.9428
10	8	0.03	0.5545	100	4	0.01	0.8431
10	8	0.035	0.4926	100	4	0.015	0.7432
10	8	0.04	0.4354	100	4	0.02	0.6487
10	8	0.045	0.3802	100	4	0.025	0.5588
10	8	0.05	0.3290	100	4	0.03	0.4789
10	10	0.001	0.9776	100	4	0.035	0.4120
10	10	0.005	0.8931	100	4	0.04	0.3531
10	10	0.01	0.7934	100	4	0.045	0.2934
10	10	0.015	0.7009	100	4	0.05	0.2469
10	10	0.02	0.6144	100	6	0.001	0.9894
10	10	0.025	0.5339	100	6	0.005	0.9407
10	10	0.03	0.4585	100	6	0.01	0.8597
10	10	0.035	0.3874	100	6	0.015	0.7720
10	10	0.04	0.3218	100	6	0.02	0.6827
10	10	0.045	0.2600	100	6	0.025	0.6000
10	10	0.05	0.2030	100	6	0.03	0.5205
10	20	0.001	0.9529	100	6	0.035	0.4539
10	20	0.005	0.7796	100	6	0.04	0.3837
10	20	0.01	0.5868	100	6	0.045	0.3278
10	20	0.015	0.4198	100	6	0.05	0.2741
10	20	0.02	0.2741	100	8	0.001	0.9853
10	20	0.025	0.1456	100	8	0.005	0.9176
10	20	0.03	0.0342	100	8	0.01	0.8179
20	4	0.001	0.9927	100	8	0.015	0.7209
20	4	0.005	0.9633	100	8	0.02	0.6314
20	4	0.01	0.9263	100	8	0.025	0.5434
20	4	0.015	0.8887	100	8	0.03	0.4671
20	4	0.02	0.8492	100	8	0.035	0.3886
20	4	0.025	0.8094	100	8	0.04	0.3269
20	4	0.03	0.7707	100	8	0.045	0.2609
20	4	0.035	0.7306	100	8	0.05	0.2076
20	4	0.04	0.6930	100	10	0.001	0.9815
20	4	0.045	0.6550	100	10	0.005	0.8940

20	4	0.05	0.6162	100	10	0.01	0.7750
20	6	0.001	0.9887	100	10	0.015	0.6681
20	6	0.005	0.9448	100	10	0.02	0.5695
20	6	0.01	0.8916	100	10	0.025	0.4827
20	6	0.015	0.8397	100	10	0.03	0.4012
20	6	0.02	0.7893	100	10	0.035	0.3272
20	6	0.025	0.7393	100	10	0.04	0.2592
20	6	0.03	0.6910	100	10	0.045	0.1966
20	6	0.035	0.6445	100	10	0.05	0.1414
20	6	0.04	0.5984	100	20	0.001	0.9609
20	6	0.045	0.5537	100	20	0.005	0.7740
20	6	0.05	0.5109	100	20	0.01	0.5706
20	8	0.001	0.9840	100	20	0.015	0.4042
20	8	0.005	0.9232	100	20	0.02	0.2707
20	8	0.01	0.8492	100	20	0.025	0.1580
20	8	0.015	0.7781	100	20	0.03	0.0614

Table S11. Decoding speed of MEPCAL with different combinations of parameters. 2,400 DNA symbols were decoded for each condition, whose average decoding speed was shown below. The DNA sequence used for decoding was randomly generated.

Decoding time (KB/s)	*S*=5	*S*=10	*S*=20	*S*=40	*S*=100
R=4	194.67	157.55	122.42	97.87	53.05
R=6	148.50	181.58	151.46	120.21	58.68
R=8	111.63	124.37	94.43	69.49	28.77
R=10	115.72	123.93	100.47	69.75	29.11
R=20	51.16	44.60	41.72	29.20	11.94

Table S12. Summary of statistics used in this study.

Position	Sample size	Number of biological replicates	Test / Fitting	p / R^2 value
Table 1	For MEPCAL-encoded sequence, 6,908 reads in nanopore sequencing (33,947,519bp in total); For natural DNA, 353,748 reads in nanopore sequencing (2,419,277,016bp in total)	/	Two-sided Kolmogorov-Smirnov test	For each case of test (distributions of substitution, insertion, deletion, all errors), $p < 0.01$
Figure 2c	20 PCR reactions for each culture condition	/	/	/
Figure 2d	/	3	/	/
Figure 4a/b/c	137,254 reads in nanopore sequencing (977,967,436bp in total)	/	/	/
Figure 4d/e/f	1,652 reads in nanopore sequencing (13,731,237bp in total)	/	/	/
Figure 5a/b/c	Individual data point was shown in the figure			
Figure 5d/e/f	2,000 DNA symbols × 20,000 simulations	/	Gaussian distribution fitting (the last subgraph in Figure 5D)	/
Figure 5f (black line)	/	/	Multivariate Polynomial Fitting	R^2=0.98
Figure S2a	/	3	Fitting to 3-parameter logistic equation	/
Figure S2b	/	3	Fitting to 3-parameter logistic equation	/
Figure S5	/	3	/	/
Figure S8a/b	20,000 50-bp DNA	/	/	/

	sequences			
Figure S10	1,048,573 reads in Hi-seq (91,942,101bp in total)	/	/	/
Figure S11b	137,254 reads in nanopore sequencing	/	/	/
Figure S11c	4,054 reads in nanopore sequencing	/	/	/
Figure S12a/b	1,652 reads (13,731,237bp in total)	/	/	/
Figure S12c/d	2,898 reads (20,319,898bp in total)	/	/	/
Figure S13a/b	6,908 reads (33,947,519bp in total)	/	/	/
Figure S13c/d	3,831 reads (23,653,240bp in total)	/	/	/
Figure S14	10,000 simulations of BCH-encoded information DNA	/	/	/
Figure S15	1,050 simulations of information DNA encoding *Les Misérables*	/	/	/

Supplementary Movie: Microscopic imaging of bacterial growth

a *E. coli* TOP10, no information DNA integrated. **b** *E. coli* TOP10, with information DNA integrated. **c** *Halomonas bluephagenesis* TD01, no information DNA integrated. **d** *Halomonas bluephagenesis* TD01, with information DNA integrated.
